# Preclinical Evaluation of Labetalol for Cutaneous Melanoma Drug Repurposing: Cytotoxic Activity in A375 Cells and Mitochondria-Associated Apoptotic Signaling

**DOI:** 10.3390/medicina62091802

**Published:** 2026-09-19

**Authors:** Richard Dahma, Elena-Alina Moacă, Iasmina-Alexandra Predescu, Ana-Cristiane Dragomir, Oana-Andrada Iftode, Stela Iurciuc, Diana Haj Ali, Ioana Macaşoi, Maria Sala-Cîrtog, Iulia-Najette Crintea, Alina-Doina Tănase, Marilena Dinuţi

**Affiliations:** 1 Department of Biochemistry and Pharmacology, Faculty of Medicine, Doctoral School, Victor Babeș University of Medicine and Pharmacy, 2nd Eftimie Murgu Square, 300041 Timişoara, Romania; dahmarici1@gmail.com (R.D.); cristiane.dragomir@umft.ro (A.-C.D.); 2 University Clinic of Toxicology, Drug Industry, Management, Marketing and Dermatopharmacy, Faculty of Pharmacy, Victor Babes University of Medicine and Pharmacy, 2nd Eftimie Murgu Square, 300041 Timisoara, Romania; alina.moaca@umft.ro (E.-A.M.); iasmina-alexandra.predescu@umft.ro (I.-A.P.); diana.haj-ali@umft.ro (D.H.A.); macasoi.ioana@umft.ro (I.M.); 3 Research Centre for Pharmaco-Toxicological Evaluation, Faculty of Pharmacy, Victor Babeș University of Medicine and Pharmacy, 2nd Eftimie Murgu Square, 300041 Timișoara, Romania; 4 Emergency City Hospital Timisoara, Gheorghe Dima Street Nr. 5, 300254 Timisoara, Romania; 5 Department of Cardiology, Victor Babeş University of Medicine and Pharmacy, 2nd Eftimie Murgu Square, 300041 Timisoara, Romania; 6 Discipline of Biochemistry, Department of Biochemistry and Pharmacology, Faculty of Medicine, Victor Babeș University of Medicine and Pharmacy, 2nd Eftimie Murgu Square, 300041 Timisoara, Romania; mariasala.cirtog@umft.ro (M.S.-C.); dinuti.marilena@umft.ro (M.D.); 7 Emergency Clinical Municipal Hospital Timisoara, Blvd. Regele Mihai I, No. 5, 300079 Timişoara, Romania; crintea.iulia@umft.ro; 8 University Clinic of Emergency, Department of Surgery I, Faculty of Medicine, Victor Babes University of Medicine and Pharmacy, 2nd Eftimie Murgu Square, 300041 Timisoara, Romania; 9 Discipline of Professional Legislation in Dentistry, Faculty of Dental Medicine, Victor Babes University of Medicine and Pharmacy, 2nd Eftimie Murgu Square, 300041 Timişoara, Romania; tanase.alina@umft.ro; 10 Methodological and Research Center Medical Law, Medical Malpractice and Research Ethics, Victor Babeș University of Medicine and Pharmacy, 2nd Eftimie Murgu Square, 300041 Timișoara, Romania

**Keywords:** labetalol, drug repurposing, melanoma, A375 cells, β-adrenergic antagonists, mitochondrial apoptosis, HET-CAM

## Abstract

*Background and Objectives*: Drug repurposing provides an opportunity to identify anticancer activities among established pharmacological agents. Labetalol (LB), an α_1_- and non-selective β-adrenergic receptor antagonist, has been insufficiently investigated in melanoma. This study evaluated the potential anti-melanoma activity of LB in A375 human melanoma cells, compared its effects with those observed in HaCaT immortalized non-tumoral keratinocytes, and investigated the cellular mechanisms associated with LB-induced cytotoxicity. *Materials and Methods*: A375 and HaCaT cells were exposed to 75–500 μM LB for 24 h. Cell viability and lysosomal dye retention were assessed using MTT and NRU assays. A DMSO-only concentration series (0.075–0.50% *v*/*v*) was additionally evaluated by MTT in both cell lines. Mitochondrial membrane potential, mitochondrial staining patterns, nuclear morphology, cytoskeletal organization, caspase-3/7 and caspase-9 activities, and plasma membrane integrity were evaluated using JC-1, MitoTracker Red CMXRos, immunofluorescence, luminescence-based caspase assays, and AO/PI staining. The acute irritation potential of 500 μM LB was assessed using the HET-CAM assay. *Results*: DMSO alone did not significantly reduce viability over the investigated concentration range in either cell line. LB reduced A375 cell viability in a concentration-dependent manner, with a 24 h IC_50_ of 422.5 μM and a viability of 33.61% at 500 μM. Under the same experimental conditions, HaCaT viability remained at 89.01% at 500 μM, and an IC_50_ was not reached within the investigated concentration range. At the highest concentration, neutral red uptake and the JC-1 aggregate/monomer ratio decreased to approximately 33% and 24% of the corresponding control values, respectively. The apoptotic index increased from approximately 3% in control cells to 31%, while caspase-3/7 and caspase-9 activities increased to approximately 482% and 324% of the control. Qualitative imaging demonstrated mitochondrial staining redistribution, cytoskeletal disorganization, apoptosis-associated morphology, and loss of plasma membrane integrity at higher concentrations. In the HET-CAM assay, 500 μM LB produced a mean irritation score of 0.71 ± 0.27, within the non-irritant range. *Conclusions*: LB displayed concentration-dependent in vitro cytotoxicity in A375 melanoma cells, whereas HaCaT keratinocytes showed limited changes under the investigated conditions. LB treatment was also associated with mitochondria-associated apoptotic signaling in A375 cells. These findings provide preliminary evidence supporting further investigation of LB within a melanoma-directed drug-repurposing strategy. Additional studies are required to establish receptor dependence, achievable local exposure, safety, and translational relevance.

## 1. Introduction

Cutaneous melanoma is an aggressive malignancy characterized by marked biological heterogeneity and a pronounced capacity for metastatic dissemination. Clinical outcome remains closely associated with established pathological variables, including Breslow thickness, ulceration, mitotic activity, lymph-node involvement, and disease stage [1,2]. Whereas localized melanoma can often be managed effectively by surgical excision, prognosis deteriorates considerably once regional or distant dissemination has occurred, emphasizing both the importance of early detection and the continuing therapeutic challenge posed by advanced disease [3,4,5]. The treatment landscape of advanced melanoma has changed substantially with the introduction of immune checkpoint inhibitors and, in molecularly selected patients, therapies targeting the BRAF/MEK signaling axis. These approaches can induce durable responses and have improved long-term outcomes; however, their benefits are not universal. Primary or acquired resistance, intratumoral heterogeneity, immune escape, treatment-related toxicity, and the absence of sufficiently robust predictive biomarkers continue to limit durable disease control [6,7,8,9]. These limitations support the continued investigation of complementary therapeutic strategies capable of acting on tumor-cell vulnerabilities and on signaling pathways that contribute to melanoma progression.

Drug repurposing, the evaluation of an established drug for an indication distinct from that for which it was originally developed, offers one such strategy. Because repurposed agents have pre-existing information regarding their formulation, pharmacology, pharmacokinetics, and safety in their approved indications, they may enter translational evaluation more rapidly and with lower early-development risk than entirely new chemical entities [10,11]. This approach is particularly attractive in oncology, where pharmacologically diverse agents may affect cancer-relevant pathways through their primary targets, additional off-target actions, or both. Nevertheless, prior clinical use does not establish anticancer efficacy, and differences between conventional therapeutic exposure and the concentrations required for antitumor activity necessitate rigorous mechanistic, pharmacokinetic, and safety assessment [10,12].

Adrenergic signaling has emerged as a potential link between systemic stress responses and tumor biology. Catecholamines acting through adrenergic receptors can influence cancer-cell proliferation, survival, migration, angiogenesis, and interactions with stromal and immune components of the tumor microenvironment [13]. Functional β-adrenergic receptor subtypes have been identified in melanoma cells and in cells of the melanoma microenvironment, providing a biological rationale for investigating β-adrenergic antagonists in this malignancy [4,14]. Preclinical studies further indicate that β-adrenergic blockade can attenuate pro-tumorigenic signaling, reduce melanoma growth and dissemination, and modify the immune and vascular microenvironment, although the relative contribution of individual receptor subtypes and receptor-independent mechanisms remains incompletely defined [4,14]. Among β-blockers, propranolol has received the greatest attention as a candidate for repurposing in melanoma. It has been reported to reduce the viability of A375 and primary acral melanoma cells, induce cell-cycle arrest and apoptosis, and inhibit AKT/MAPK-associated signaling [15]. More recent comparative evidence in A375 cells showed concentration-dependent reductions in viability with the non-selective β-blockers carvedilol and propranolol, whereas the β_1_-selective antagonists atenolol and metoprolol produced no significant viability reduction under the same experimental conditions [16]. Clinical and epidemiological findings remain less consistent: some reports suggest delayed melanoma progression or improved outcomes during β-blocker exposure, whereas other population-based analyses have found no significant association with melanoma-specific survival [4,17]. Collectively, these observations indicate that β-blockers should not be regarded as a pharmacologically uniform anticancer class and that each agent requires drug-specific evaluation.

Labetalol (LB) is an established antihypertensive agent that combines α_1_-adrenergic receptor antagonism with non-selective β-adrenergic receptor blockade [18]. Despite this pharmacological profile, which could theoretically interfere with multiple components of adrenergic signaling, its potential anticancer activity has received limited investigation. In a murine epidermal JB6 P+ model, labetalol inhibited epidermal growth factor-induced neoplastic transformation at concentrations that produced little cytotoxicity, suggesting an effect on transformation-related signaling rather than nonspecific cell killing in that system [19]. Labetalol has also been included in comparative studies of non-selective β-blockers in three-dimensional uveal melanoma spheroids, in which activity was observed at higher micromolar concentrations, although carvedilol was the most potent agent and was selected for detailed mechanistic evaluation [20]. Because uveal and cutaneous melanomas are biologically distinct, these findings cannot establish the activity of labetalol in cutaneous melanoma. Moreover, direct information regarding its effects in A375 cells, its relative impact on melanoma and non-tumoral skin-derived cells, and the cellular events associated with any cytotoxic response remains scarce. Given the potential for local drug delivery in melanoma, locally applied LB formulations may represent a future translational direction, potentially as an adjunct to established therapies rather than as a replacement for standard treatment.

Within this drug-repurposing framework, the present study aimed to investigate the potential anti-melanoma activity of LB in A375 human melanoma cells and to compare its effects on cellular viability and morphology with those observed in HaCaT immortalized non-tumoral keratinocytes. To characterize the cellular response, complementary assays were used to examine lysosomal activity, mitochondrial membrane potential and morphology, nuclear and cytoskeletal organization, caspase activation, and morphological features of cell death. In addition, the acute irritation potential of an LB-containing test solution was preliminarily assessed using the hen’s egg test–chorioallantoic membrane (HET-CAM) model. This experimental design was intended to provide an initial evaluation of LB as a melanoma-directed repurposing candidate and to identify cellular processes that warrant further mechanistic and translational investigation.

## 2. Materials and Methods

### 2.1. Reagents and Instruments

Labetalol (LB), phosphate-buffered saline (PBS), Acridine Orange, Propidium Iodide, and Triton X-100 were supplied by Sigma-Aldrich (Merck KGaA, Darmstadt, Germany). Cell culture reagents, including Dulbecco’s Modified Eagle’s Medium (DMEM), fetal bovine serum (FBS), and penicillin/streptomycin solution (100 U/mL–100 μg/mL), were obtained from PAN-Biotech GmbH (Aidenbach, Germany). The MTT Cell Proliferation Assay Kit (3-(4,5-dimethylthiazol-2-yl)-2,5-diphenyltetrazolium bromide) was purchased from Roche (Welwyn Garden City, UK). Reagents used for fluorescence-based analyses, namely Hoechst 33342, MitoTracker^TM^ Red CMXRos, Texas Red^TM^-X Phalloidin, the anti-α-tubulin monoclonal antibody (B-5-1-2), and the Alexa Fluor^TM^ 488 conjugated goat anti-mouse IgG (H+L) secondary antibody, were acquired from Thermo Fisher Scientific (Waltham, MA, USA). The JC-1 Mitochondrial Membrane Potential Assay Kit was sourced from Elabscience (Houston, TX, USA). A ready-to-use 4% paraformaldehyde solution prepared in PBS was purchased from Santa Cruz Biotechnology (Dallas, TX, USA), while bovine serum albumin (BSA) was obtained from Cell Signaling Technology (Danvers, MA, USA). The Caspase-Glo^®^ 3/7 and Caspase-Glo^®^ 9 assay kits were supplied by Promega Corporation (Madison, WI, USA).

The in vitro experiments were performed using a Cytation 5 multimodal microplate reader and a Lionheart FX automated microscope (BioTek Instruments, Winooski, VT, USA), and data analysis was conducted using Gen5^TM^ Microplate Data Collection and Analysis software (version 3.14) provided by BioTek Instruments Inc. (Winooski, VT, USA). The in ovo experiment was conducted using a Zeiss Stereo Discovery.V8 stereomicroscope (Zeiss, Göttingen, Germany) equipped with an AxioCam 105 color camera.

### 2.2. Cell Culture Protocol

The experiments were conducted using the human melanoma cell line A375 (CRL-1619^TM^) purchased from American Type Culture Collection (ATCC) (Manassas, VA, USA), and the immortalized human keratinocyte cell line HaCaT (300493) sourced from Cell Lines Service (CLS) (Eppelheim, Germany). A375 cells were used to evaluate the antitumor activity of LB, while HaCaT cells were used to evaluate the safety profile of the compound. Both cell lines were cultured in Dulbecco’s Modified Eagle’s Medium (DMEM) supplemented with 10% fetal bovine serum (FBS) and 1% penicillin–streptomycin solution and maintained in a humidified incubator at 37 °C with 5% CO_2_.

An LB stock solution (100 mM) was prepared in dimethyl sulfoxide (DMSO) and subsequently diluted in the appropriate culture medium to obtain final LB concentrations of 75, 150, 175, 300, 400, 450, and 500 μM. The corresponding final DMSO concentrations were 0.075%, 0.150%, 0.175%, 0.30%, 0.40%, 0.45%, and 0.50% (*v*/*v*), respectively. Thus, the final DMSO concentration did not exceed 0.50% (*v*/*v*) under any experimental condition. Untreated cells maintained in culture medium were used as controls. Data from the literature indicate that DMSO concentrations within the range of 0.10–1% (*v*/*v*) are generally considered acceptable for most cell-based assays, although cellular responses may vary depending on the cell line and experimental conditions [21,22]. Moreover, studies performed specifically on the A375 and HaCaT cell lines support the tolerability of DMSO at concentrations of up to 0.50% (*v*/*v*) under the investigated exposure time [23,24].

The concentration range evaluated in this study was selected based on previous in vitro investigations reporting antitumor effects of β-adrenergic receptor antagonists in melanoma models. Since the activity of LB in cutaneous melanoma cells has not been previously characterized, a broad concentration range was intentionally employed to assess concentration-dependent effects and to determine whether the 50% viability threshold could be reached. Previous studies in A375 melanoma cells have likewise used high micromolar concentrations of β-blockers, extending up to 400–500 µM for some agents, while LB itself has been evaluated at high micromolar concentrations in uveal melanoma spheroids [16]. Accordingly, the upper concentration of 500 µM was selected to provide a broader characterization of the LB concentration–response profile.

### 2.3. Cell Viability Assessment—The MTT Test

The cell viability of A375 and HaCaT cells was evaluated using the MTT (3-(4,5-dimethylthiazol-2-yl)-2,5-diphenyltetrazolium bromide) assay, according to a protocol described by Vicaș et al. [25]. Thus, the cells were cultured in 96-well plates (1 × 10^4^ cells/well), and when optimal confluence was reached, they were treated for 24 h with LB at concentrations ranging from 75 to 500 μM. At the end of the treatment, the culture medium was replaced with fresh medium, and 10 µL of MTT was added to each well. The plates were then incubated for 3 h at 37 °C and 5% CO_2_. Subsequently, 100 µL of MTT solubilizing solution was added to each well, and the plates were incubated at room temperature for 30 min, and absorbance was measured at 570 nm and 630 nm using the Cytation 5 microplate reader.

To further evaluate the potential contribution of the vehicle to the observed viability response, an additional DMSO-only experiment was performed in both A375 and HaCaT cells. Cells were exposed for 24 h to the exact final DMSO concentrations corresponding to the LB treatment series: 0.075%, 0.150%, 0.175%, 0.30%, 0.40%, 0.45%, and 0.50% (*v*/*v*). Cell viability was subsequently assessed using the same MTT protocol described above. Untreated cells maintained in culture medium were used as the reference control.

The IC_50_ value was calculated using GraphPad Prism software, version 11.0.2 (GraphPad Software, San Diego, CA, USA, www.graphpad.com), by nonlinear regression analysis, fitting the concentration–response data to the “log(inhibitor) vs. response—Variable slope (four parameters)” model. The IC_50_ value is reported with its corresponding 95% confidence interval (95% CI).

### 2.4. Cell Morphology Assessment

Bright-field microscopy was used to assess any morphological changes in A375 and HaCaT cells after 24 h of treatment with LB at concentrations ranging from 75 to 500 μM. Representative images were obtained at the end of the treatment period using a Lionheart FX automated imaging system.

### 2.5. Neutral Red Staining and Uptake

A375 cells were seeded in 96-well culture plates at a density of 1 × 10^4^ cells per well using DMEM supplemented with 10% FBS. After cell attachment, A375 cells were exposed to LB at concentrations ranging from 75 to 500 µM for 24 h. Following treatment, the medium was removed and replaced with 100 µL of neutral red working solution (40 µg/mL in DMEM) per well. The plates were then incubated for 2 h at 37 °C in a humidified atmosphere containing 5% CO_2_. Afterwards, cells were washed with PBS (150 µL/well), and representative bright-field images were acquired using a Lionheart FX automated imaging system. To extract the accumulated dye, 150 µL/well of a destaining solution consisting of approximately 50% ethanol, 49% ultrapure water, and 1% glacial acetic acid was added. The absorbance of the resulting extracts was measured at 540 nm using a Cytation 5 microplate reader, allowing quantification of neutral red uptake. The assay protocol was adapted from the methodology described by Repetto et al. [26].

### 2.6. Mitochondrial Membrane Potential (ΔΨm) Assay—JC-1 Staining

Changes in mitochondrial membrane potential (ΔΨm) induced by LB exposure in A375 cells were evaluated using the JC-1 fluorescent probe assay, following the manufacturer’s instructions. Cells were seeded into black-walled, clear-bottom 96-well plates at a density of 1 × 10^4^ cells per well and allowed to grow until approximately 70% confluence was reached. Subsequently, cells were exposed to LB at concentrations of 75–500 µM for a period of 24 h. After treatment, the culture medium was discarded, and the cells were carefully washed with PBS. The cells were then incubated with JC-1 staining solution (5 µM, 100 µL/well) for 45 min at 37 °C. Upon completion of the staining step, excess dye was removed by washing the cells twice with PBS. Fluorescence images were acquired using a Lionheart FX automated imaging system and further analyzed with Gen5^TM^ Microplate Data Collection and Analysis Software (v3.14). Fluorescence emissions corresponding to JC-1 aggregates (red fluorescence, 590 nm) and monomers (green fluorescence, 529 nm) were measured using a Cytation 5 multimode microplate reader (BioTek Instruments, USA). Mitochondrial membrane potential was assessed by calculating the ratio of red to green fluorescence signals. The resulting values were normalized against untreated control cells, which were assigned a value of 100%, and the data were expressed as percentages relative to the control group. The experimental protocol was similar to that described by Talpoș et al. [27].

### 2.7. Mitochondria Fluorescence Staining

MitoTracker^TM^ Red CMXRos fluorescence staining was used for the qualitative visualization of mitochondrial distribution and morphology in A375 cells. A375 cells were cultured in 12-well plates (1 × 10^5^ cells/well) and, once 70% confluence was reached, the cells were treated for 24 h with LB at concentrations ranging from 75 to 500 μM. Following treatment, a MitoTracker^TM^ Red CMXRos staining solution was prepared by dissolving the dye in DMSO to obtain a 1 mM stock solution, which was subsequently diluted in the appropriate culture medium to a final concentration of 300 nM. The cells were then incubated with the staining solution for 30–45 min at 37 °C in a humidified atmosphere containing 5% CO_2_. After incubation, the cells were washed three times with PBS, and images were acquired using a Lionheart FX automated microscope [28].

### 2.8. Immunofluorescence Staining of Nuclei, Tubulin and F-Actin Filaments

Immunofluorescence analysis was used to evaluate the effects of 24 h exposure to LB at concentrations ranging from 75 to 500 μM on nuclear architecture as well as on the organization of F-actin and tubulin filaments in A375 cells. For this purpose, cells were seeded in black-walled, clear-bottom 96-well plates at a density of 1 × 10^4^ cells per well and allowed to adhere until reaching the desired confluence. Subsequently, the cultures were treated with LB. Following treatment, cells were fixed with 4% paraformaldehyde for 15 min and washed twice with PBS. Membrane permeabilization was then performed using 0.1% Triton X-100 (50 μL/well) for 15 min at room temperature, followed by two additional PBS washes. To minimize nonspecific antibody binding, cells were incubated with 1% bovine serum albumin (BSA; 100 μL/well) for 30 min at room temperature. Tubulin was visualized by incubating the samples with an anti-α-tubulin monoclonal antibody (B-5-1-2) diluted 1:500 in 1% BSA (50 μL/well) for 3 h. Then, cells were incubated for 45 min with an Alexa Fluor^TM^ 488-conjugated goat anti-mouse IgG (H+L) secondary antibody prepared at a 1:500 dilution. Following two PBS washing steps, a staining solution containing phalloidin Texas Red^TM^-X (1:200 dilution) for F-actin detection and Hoechst 33342 (1:2000 dilution) for nuclear labeling was added (50 μL/well). The staining procedure was carried out for 30 min at room temperature in the dark. At the end of the protocol, cells were washed twice with PBS, and fluorescence images were captured using a Lionheart FX Automated Microscope. Image acquisition and processing were performed with Gen5^TM^ Microplate Data Collection and Analysis Software (Version 3.14, BioTek Instruments, USA). The methodology was adapted from a previously reported immunofluorescence protocol described by Marcovici et al. [29].

Nuclei exhibiting apoptotic morphological features, including chromatin condensation and/or nuclear fragmentation, were classified as apoptotic. The apoptotic index was calculated according to the following formula:

$$Apoptot\;icindex\left(\%\right)=\frac{no.\;of\;apoptotic\;nuclei}{total\;no.\;of\;nuclei\;evaluated}\times 100$$

### 2.9. Caspase 3/7 and Caspase 9 Activation

The potential activation of caspase-3/7 and caspase-9 in response to LB treatment was assessed using caspase-Glo^®^ assays. A375 cells were seeded in white opaque 96-well microplates at a density of 1 × 10^4^ cells per well and allowed to adhere under standard culture conditions. Subsequently, the cells were exposed to LB at concentrations of 75–500 µM for 24 h. At the end of the 24 h treatment period, the plates were equilibrated to room temperature. Caspase-Glo^®^ 3/7 or Caspase-Glo^®^ 9 reagent was prepared according to the manufacturer’s instructions and added directly to each well in a volume equal to that of the culture medium. The plates were mixed for 30 s on an orbital shaker and incubated at room temperature for 3 h under light-protected conditions. Luminescence was subsequently measured using a Cytation 5 multimode microplate reader (BioTek Instruments, USA). Following subtraction of the reagent-only background signal, the obtained values were normalized to the untreated control, which was assigned a value of 100%. Three independent experiments were performed, each using three technical replicates. The experimental protocol was carried out in accordance with the methodology described by Feher et al. [30].

### 2.10. Acridine Orange/Propidium Iodide (AO/PI) Assay

To assess membrane integrity and viability, a double-staining assay with acridine orange/propidium iodide (AO/PI) was performed. A375 cells were seeded in 96-well plates (1 × 10^4^ cells/well) and treated for 24 h with LB at concentrations ranging from 75 to 500 μM. At the end of the treatment period, an AO/PI staining solution containing acridine orange (10 μg/mL) and propidium iodide (10 μg/mL) in culture medium was prepared, and 100 μL of this staining solution was added to each well. The plates were then incubated for 10 min at room temperature in the dark. Fluorescence images were captured using a Lionheart FX automated microscope and analyzed using Gen5^TM^ Microplate Data Collection software.

### 2.11. HET-CAM Assay

The irritant potential of LB was evaluated using the Hen’s Egg Test—chorioallantoic membrane (HET-CAM) model. Fertilized hen’s eggs (*Gallus gallus domesticus*) were disinfected with 70% ethanol and incubated at 37 °C under controlled humidity conditions. On the fourth day of incubation, a small cut was made in the eggshell, and approximately 6–7 mL of albumen was carefully removed to facilitate separation of the chorioallantoic membrane (CAM) from the shell. The cut was sealed with adhesive tape, and the eggs were returned to the incubator. On day five, a window was opened in the top of the eggshell to expose the CAM and allow direct visualization of its vascular network. The window was then covered with adhesive tape, and incubation continued until the day of testing. The experiment was conducted on day nine. LB was evaluated at a concentration of 500 μM by applying 600 μL of the sample directly to the CAM surface. Distilled water, used as the vehicle for LB preparation, served as the negative/vehicle control, while 1% SLS was used as the positive control. All experimental conditions were performed in triplicate. After treatment, the vascular response of the CAM was monitored for 5 min to observe potential alterations in the blood vessels, such as hemorrhage (H), lysis (L), and coagulation (C). For reactions not observed within the 300 s monitoring period, an onset time of 301 s was assigned for IS calculation. Representative images were obtained immediately before exposure (T_0_) and at the end of the observation period (T_5_) using a Stereo Discovery.V8 stereomicroscope equipped with an AxioCam 105 color camera (Zeiss, Göttingen, Germany).

The irritation score (IS) was calculated based on the onset times of H (t_H_), L (t_L_), and C (t_C_) observed during the monitoring period, according to the following equation [31]:

$$IS=5\times \frac{301-t_{H}}{300}+7\times \frac{301-t_{L}}{300}+9\times \frac{301-t_{C}}{300}$$

According to the obtained IS values, substances can be classified as non-irritant (IS = 0–0.9), irritant (IS = 1–8.9), or severely irritant (IS = 9–21) [32,33].

### 2.12. Statistical Analysis

Statistical analysis was performed using GraphPad Prism software, version 11.0.2 (GraphPad Software, San Diego, CA, USA, www.graphpad.com). Three independent experiments were performed, each in technical triplicate. Technical replicates were averaged within each independent experiment, and the independent experiment was considered the experimental unit (*n* = 3). Statistical differences between treated groups and the untreated control were analyzed using one-way ANOVA followed by Dunnett’s multiple-comparisons test. In experiments involving both A375 and HaCaT cells, each cell line was analyzed separately relative to its corresponding untreated control. All statistically significant results were marked with “*” as follows: * *p* < 0.05; ** *p* < 0.01; *** *p* < 0.001; **** *p* < 0.0001. The additional DMSO-only viability experiment was analyzed using the same statistical approach, with each DMSO concentration compared with the corresponding untreated control using one-way ANOVA followed by Dunnett’s multiple-comparisons test.

## 3. Results

### 3.1. Effects of LB on A375 Melanoma Cell and HaCaT Keratinocyte Viability

The effects of LB on the viability of A375 human melanoma cells and HaCaT immortalized human keratinocytes were evaluated using the MTT assay after 24 h of exposure. Cell viability was expressed relative to the untreated control, which was assigned a value of 100%. Data are presented as mean ± standard deviation (SD) from three independent experiments, each performed in triplicate.

**Figure 1:**
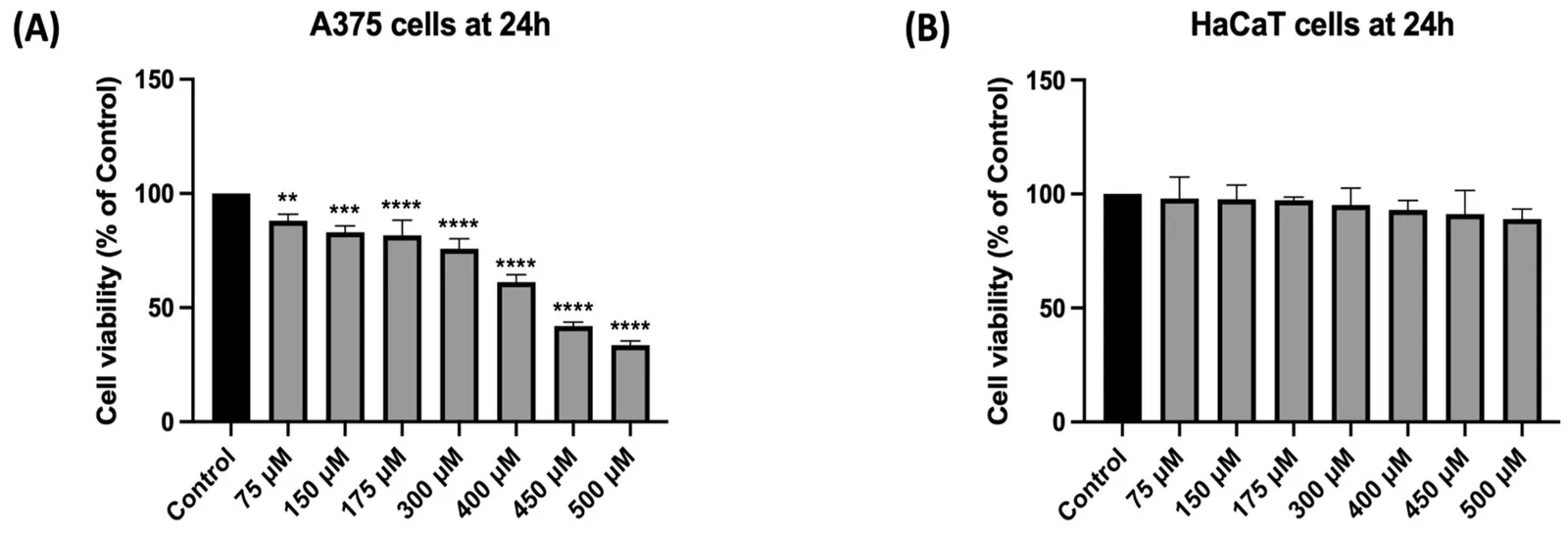
Effects of 24 h exposure to LB (75–500 µM) on the viability of (**A**) A375 malignant melanoma cells and (**B**) HaCaT immortalized human keratinocytes. Cell viability was assessed using the MTT assay and expressed as a percentage relative to the untreated control, which was assigned a value of 100%. Data are presented as mean ± standard deviation (SD) from three independent experiments, each performed in triplicate. Statistical differences between treated groups and their corresponding untreated controls were assessed using one-way ANOVA, followed by Dunnett’s multiple comparisons test. Statistical significance is indicated as follows: ** *p* < 0.01; *** *p* < 0.001; **** *p* < 0.0001.

In A375 cells (Figure 1A), LB induced a concentration-dependent reduction in cell viability. At concentrations ranging from 75 to 300 µM, the decrease was moderate, with viability declining from approximately 88% at 75 µM to approximately 76% at 300 µM. A more pronounced effect was observed at higher concentrations, with viability decreasing to approximately 61% at 400 µM and 33.61% at 500 µM. Compared with the untreated control, the reduction was statistically significant from the lowest tested concentration of 75 µM (*p* < 0.01), with increasing levels of statistical significance at 150 µM (*p* < 0.001) and from 175 µM onward (*p* < 0.0001). Based on the concentration–response curve, the half-maximal inhibitory concentration (IC_50_) after 24 h of exposure was 422.5 µM (95% CI: 382.1–480.1 µM).

In contrast, HaCaT keratinocytes (Figure 1B) cell viability gradually decreased from approximately 98% at 75 µM to 89.01% at 500 µM, without reaching statistical significance relative to the corresponding untreated control at any of the tested concentrations. Moreover, an IC_50_ value was not reached within the investigated concentration range.

To determine whether the concentration-dependent variation in DMSO content could independently affect cellular metabolic viability, A375 and HaCaT cells were additionally exposed for 24 h to the seven DMSO concentrations corresponding to the LB treatment conditions. In A375 cells, viability was 102.61 ± 6.57%, 99.30 ± 8.65%, 97.96 ± 4.53%, 98.54 ± 10.02%, 96.62 ± 1.65%, 96.16 ± 8.45%, and 94.66 ± 7.68% at 0.075%, 0.150%, 0.175%, 0.30%, 0.40%, 0.45%, and 0.50% DMSO, respectively. In HaCaT cells, the corresponding values were 104.95 ± 4.03%, 102.55 ± 4.66%, 98.79 ± 8.56%, 97.34 ± 8.86%, 94.40 ± 7.55%, 92.53 ± 8.86%, and 93.64 ± 5.32%. No statistically significant differences were observed between any DMSO-treated group and the corresponding untreated control in either cell line (*p* > 0.05). Moreover, the DMSO-only concentration series did not reproduce the pronounced concentration-dependent reduction in viability observed following LB exposure. These findings indicate that DMSO alone, within the concentration range present in the treatment solutions, was not sufficient to account for the observed MTT viability response. Given that none of the DMSO-only conditions differed significantly from the untreated control in the MTT assay, untreated cells were retained as the reference for presentation of the MTT viability data, while the DMSO-only series is reported separately as a direct assessment of the potential vehicle contribution.

### 3.2. Morphological Effects of LB on A375 and HaCaT Cells

**Figure 2:**
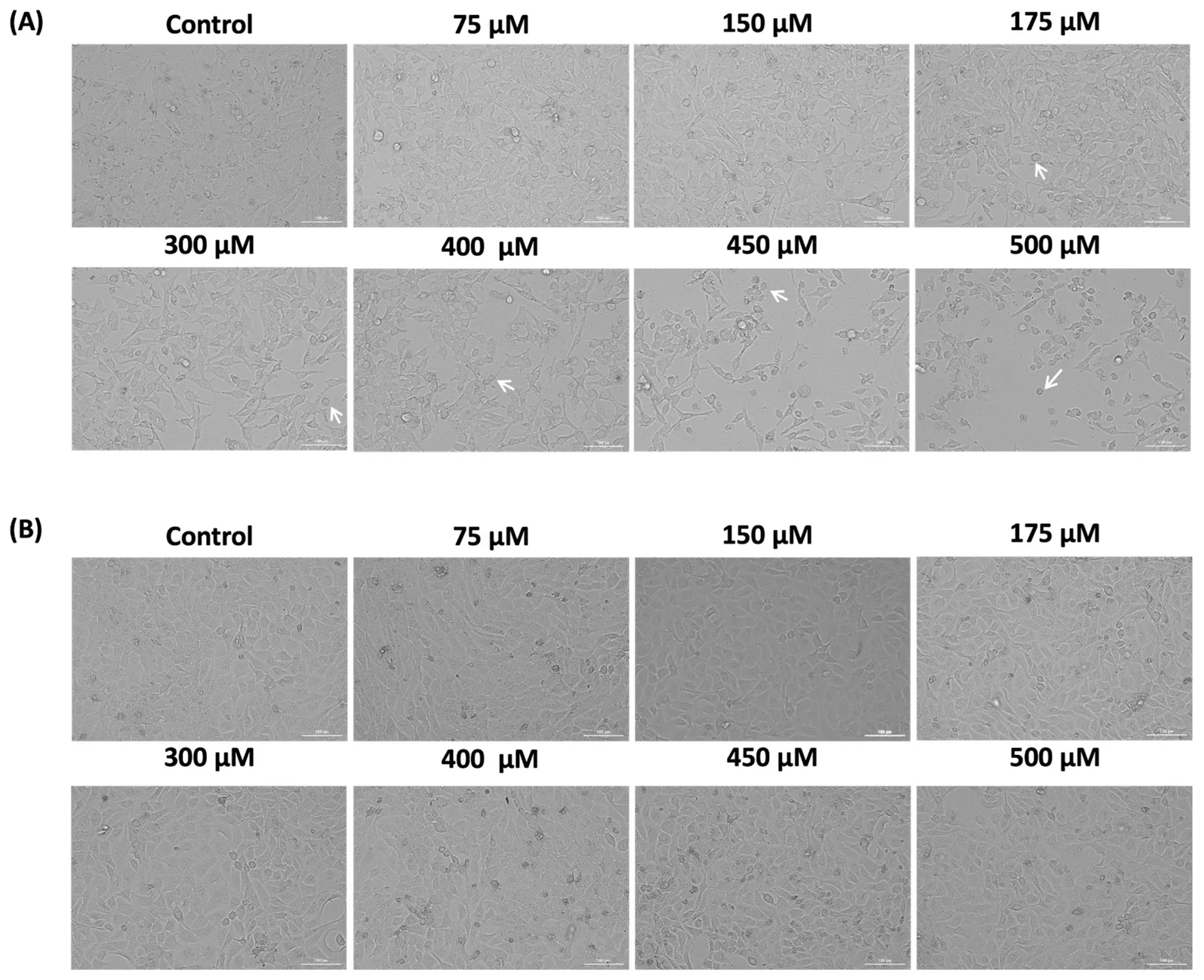
Representative bright-field images of (**A**) A375 malignant melanoma cells and (**B**) HaCaT immortalized human keratinocytes following treatment with LB (75–500 µM) for 24 h. White arrows indicate selected cells displaying morphological alterations, including rounding, shrinkage, or partial detachment. Scale bar: 100 µm.

Representative bright-field images were examined to complement the MTT findings and to qualitatively assess the morphological effects of LB after 24 h of treatment. A375 cells exposed to 75 and 150 µM LB retained an adherent morphology and cell density generally comparable to those of the untreated control. At 175 and 300 µM, early morphological changes became apparent, including an increase in rounded cells and a modest reduction in cell spreading and adherent cell density. These alterations were more evident at 400 µM and became pronounced at 450 and 500 µM, where numerous cells appeared rounded or shrunken, with partial detachment from the culture surface and a marked reduction in adherent cell density (Figure 2A). In contrast, HaCaT cells largely retained their characteristic adherent morphology throughout the investigated concentration range. A slight apparent reduction in cell density was observed at the highest concentrations, particularly at 450 and 500 µM; however, the extensive cell rounding, shrinkage, and detachment observed in A375 cultures were not evident in HaCaT cells (Figure 2B).

Overall, these qualitative observations were consistent with the MTT findings, with more pronounced LB-induced morphological alterations observed in A375 cells and comparatively limited changes in HaCaT cells under the investigated conditions.

### 3.3. LB Reduces Neutral Red Uptake in A375 Cells

Because the MTT assay primarily reflects cellular reducing activity, the neutral red uptake (NRU) assay was employed as a complementary endpoint based on the ability of viable cells to accumulate and retain neutral red within acidic lysosomal compartments.

**Figure 3:**
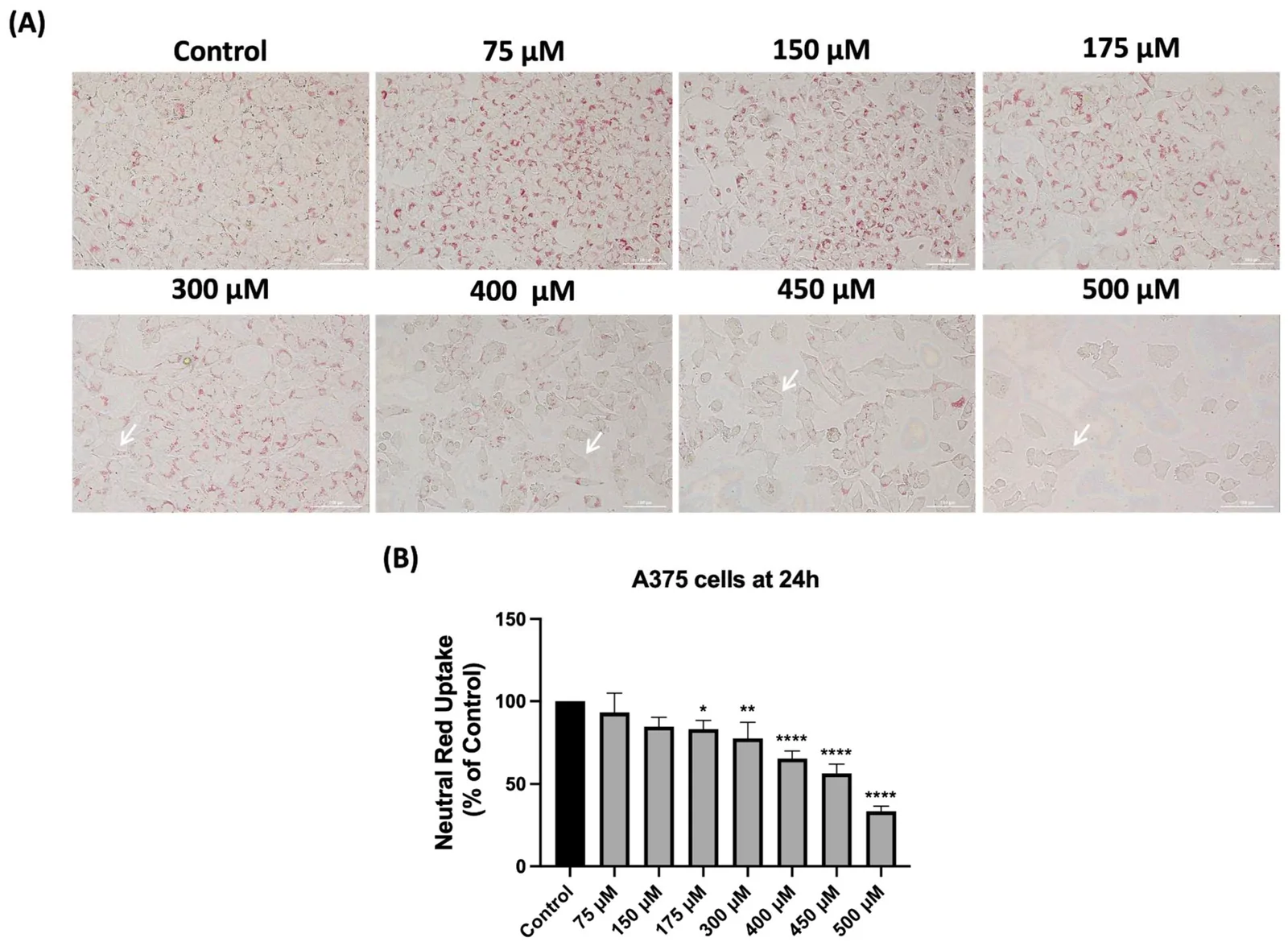
Effect of LB on neutral red uptake in A375 melanoma cells following 24 h of treatment with concentrations ranging from 75 to 500 µM. (**A**) Representative bright-field images showing intracellular neutral red accumulation. White arrows indicate representative cells or areas displaying reduced dye accumulation. (**B**) Quantification of neutral red uptake normalized to the untreated control, which was assigned a value of 100%. Data are presented as mean ± SD, and the assay was performed in triplicate. Statistical differences between the treated groups and the untreated control were determined using one-way ANOVA followed by Dunnett’s multiple-comparisons test: * *p* < 0.05, ** *p* < 0.01, and **** *p* < 0.0001. Scale bar: 100 µm.

Representative bright-field images showed a concentration-related reduction in intracellular neutral red accumulation following 24 h of LB exposure. Cells treated with 75 and 150 µM LB retained staining patterns broadly comparable to those of the untreated control. A reduction in the number and intensity of neutral-red-positive cells became progressively apparent at 175 and 300 µM and was more pronounced at 400–500 µM. The lowest intracellular dye accumulation and the greatest reduction in the density of stained adherent cells were observed at 500 µM (Figure 3A). The microscopic observations were supported by the quantitative analysis. Neutral red uptake decreased progressively from approximately 93% of the control value at 75 µM to approximately 33% at 500 µM (Figure 3B). Compared with the untreated control, the reduction reached statistical significance at 175 µM (* *p* < 0.05), 300 µM (** *p* < 0.01), and 400–500 µM (**** *p* < 0.0001).

The NRU concentration–response profile broadly paralleled that obtained using the MTT assay, particularly at the higher concentrations. These complementary findings support a concentration-dependent reduction in A375 cell viability following LB exposure. However, the decrease in NRU signal may reflect a reduction in the number of viable cells, impaired lysosomal dye uptake or retention, or a combination of these effects; therefore, the assay does not independently demonstrate a specific lysosomal mechanism.

### 3.4. LB Reduces the Mitochondrial Membrane Potential in A375 Cells

**Figure 4:**
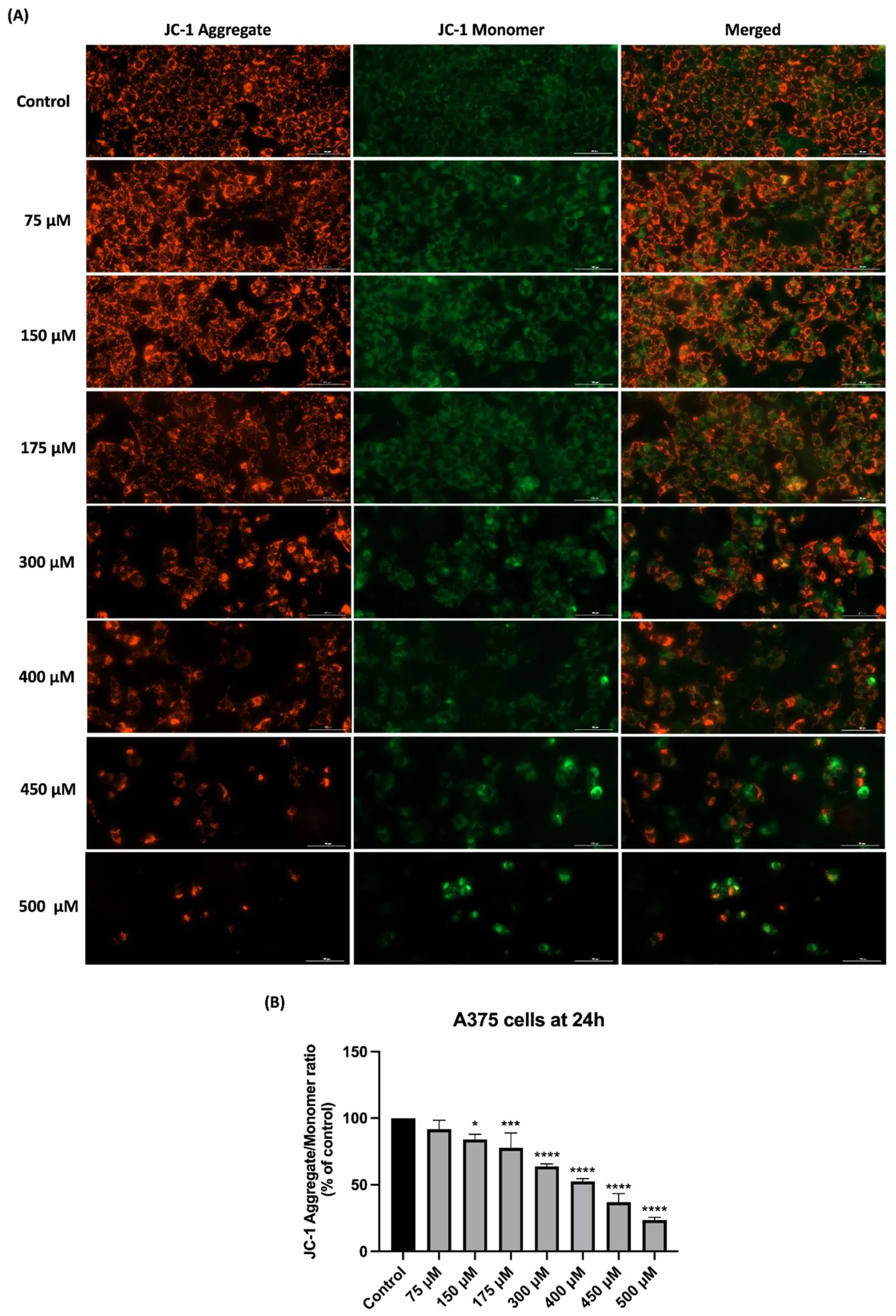
Effects of 24 h exposure to LB (75–500 µM) on the mitochondrial membrane potential of A375 cells. (**A**) Representative fluorescence images obtained following JC-1 staining. Orange–red fluorescence corresponds to JC-1 aggregates formed in polarized mitochondria, whereas green fluorescence corresponds to JC-1 monomers and is associated with mitochondrial depolarization. Merged images illustrate the concentration-dependent shift in the JC-1 fluorescence pattern. The scale bar represents 100 µm. (**B**) Quantitative analysis of mitochondrial membrane potential expressed as the JC-1 aggregate/monomer fluorescence ratio. Values were normalized to those of untreated control cells, which were assigned a value of 100%. Data are presented as mean ± SD from three independent experiments. Statistical comparisons between treated and control cells were performed using one-way ANOVA followed by Dunnett’s multiple-comparison test. * *p* < 0.05, *** *p* < 0.001, and **** *p* < 0.0001.

The effect of LB on the mitochondrial membrane potential (ΔΨm) was evaluated by JC-1 staining following 24 h of treatment. Representative fluorescence images showed a concentration-dependent shift from orange–red fluorescence, corresponding to JC-1 aggregates in polarized mitochondria, towards a relative predominance of green fluorescence, corresponding to the monomeric form of JC-1 in depolarized mitochondria (Figure 4A). These changes were most evident at the highest LB concentrations.

Quantitative analysis of the JC-1 aggregate/monomer fluorescence ratio confirmed a concentration-dependent reduction in ΔΨm (Figure 4B). The normalized ratio decreased from approximately 92% at 75 µM to approximately 24% at 500 µM. Compared with untreated control cells, the reduction was statistically significant at 150 µM (* *p* < 0.05), became more pronounced at 175 µM (*** *p* < 0.001), and was highly significant at concentrations ranging from 300 to 500 µM (**** *p* < 0.0001).

Overall, these findings demonstrate that 24 h exposure to LB induces a concentration-dependent loss of mitochondrial membrane potential in A375 melanoma cells.

### 3.5. Qualitative Alterations in MitoTracker Red CMXRos Staining Following LB Treatment

**Figure 5:**
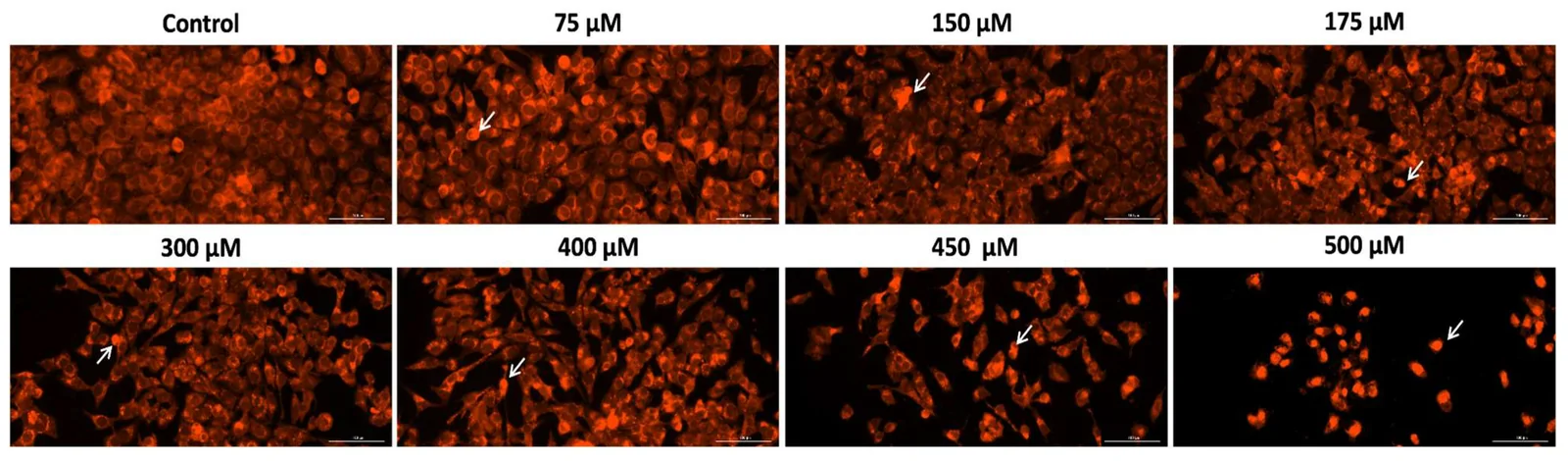
Representative MitoTracker^TM^ Red CMXRos fluorescence images of A375 cells following 24 h exposure to LB at concentrations ranging from 75 to 500 µM. Images were acquired using identical acquisition settings. Increasing LB concentrations were accompanied by changes in cellular morphology and in the intracellular distribution of the MitoTracker-positive fluorescence signal, particularly at 450 and 500 µM. White arrows indicate examples of cells displaying a compact or discontinuous mitochondrial staining pattern and altered cellular morphology. The images provide a qualitative assessment and were not subjected to fluorescence intensity or mitochondrial network morphometric analysis. The scale bar represents 100 µm.

To qualitatively examine the effects of LB on mitochondrial distribution and cellular morphology, A375 cells were stained with MitoTracker^TM^ Red CMXRos following 24 h of treatment (Figure 5). Representative images were acquired using identical acquisition settings for all experimental groups.

Untreated cells formed a dense monolayer and displayed a relatively uniform distribution of the MitoTracker-positive signal throughout the cytoplasm. Exposure to increasing concentrations of LB was accompanied by progressive changes in cellular morphology and in the distribution of the mitochondrial fluorescence signal. At the higher concentrations, particularly 450 and 500 µM, the cultures displayed reduced cell density, an increased number of rounded or contracted cells, and a more compact and discontinuous distribution of the MitoTracker-positive signal.

These qualitative observations indicate that LB-induced cellular injury is accompanied by alterations in the intracellular distribution of the mitochondrial staining pattern. However, because fluorescence intensity and mitochondrial network parameters were not quantitatively assessed, the images cannot independently establish an increase in mitochondrial fluorescence or distinguish between mitochondrial fission, fusion, aggregation, and other forms of mitochondrial structural alteration.

### 3.6. LB Induces Nuclear and Cytoskeletal Alterations in A375 Cells

**Figure 6:**
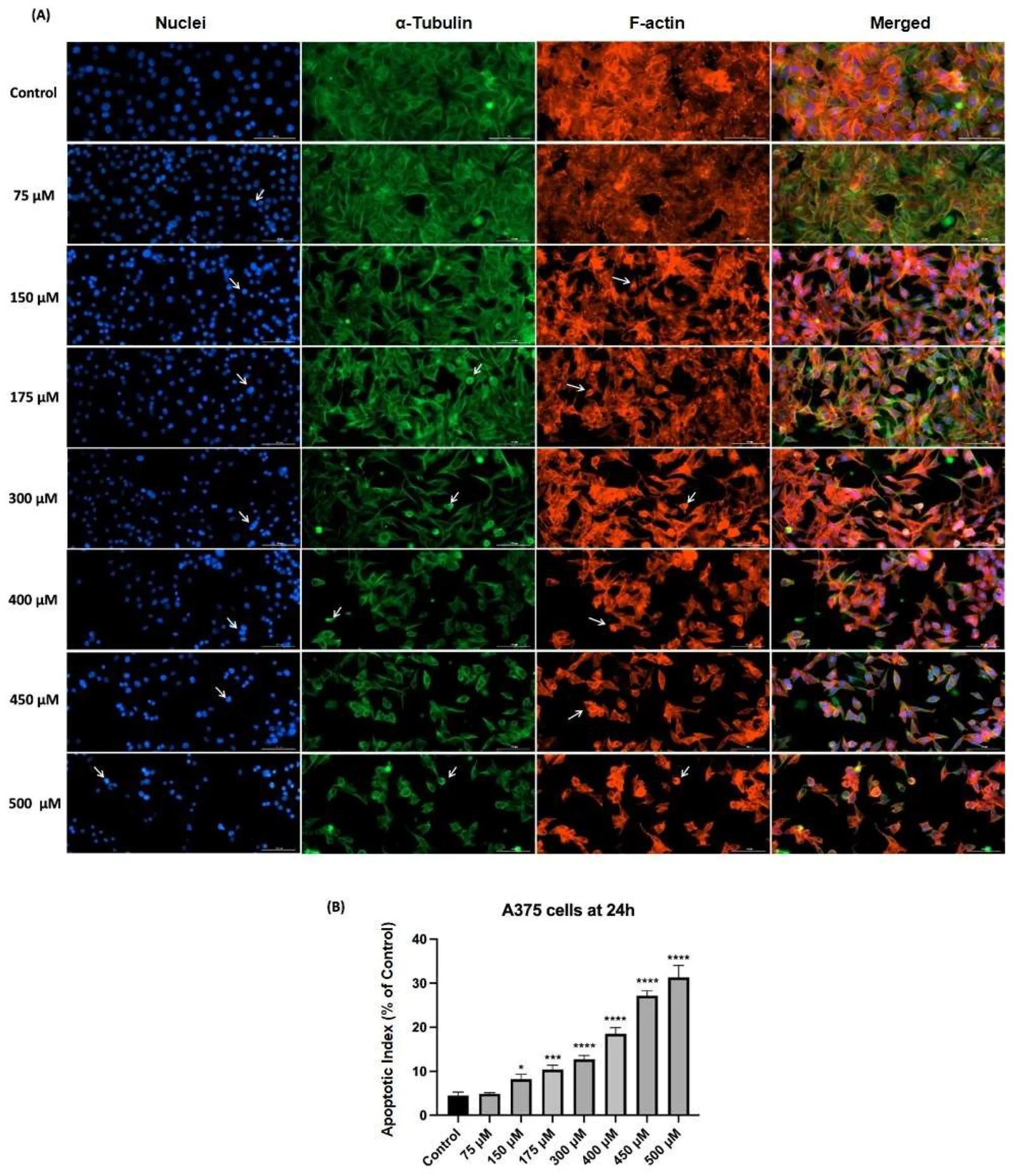
Effects of LB on nuclear morphology and cytoskeletal organization in A375 cells. Cells were exposed to LB at concentrations ranging from 75 to 500 μM for 24 h. (**A**) Representative fluorescence images showing Hoechst 33342-stained nuclei (blue), α-tubulin (green; Alexa Fluor 488), F-actin (red; Texas Red-X phalloidin), and the corresponding merged images. White arrows indicate representative cells displaying apoptotic nuclear morphology and associated cytoskeletal alterations. The scale bar indicates 100 μm. (**B**) Apoptotic index, expressed as the percentage of nuclei displaying apoptotic morphology among the total number of nuclei evaluated. Data are presented as mean ± standard deviation (SD) from three independent experiments. Statistical comparisons between treated and untreated cells were performed using one-way ANOVA followed by Dunnett’s multiple-comparison test. Statistical significance is indicated as follows: * *p* < 0.05; *** *p* < 0.001; **** *p* < 0.0001.

The effects of LB on nuclear morphology and cytoskeletal organization were evaluated after 24 h of exposure to concentrations ranging from 75 to 500 μM. Untreated A375 cells displayed largely uniform nuclear morphology and organized α-tubulin and F-actin networks (Figure 6A). In contrast, LB treatment resulted in a concentration-dependent increase in nuclei exhibiting apoptotic morphological features, particularly chromatin condensation and nuclear fragmentation. These alterations became increasingly evident at the higher concentrations.

Quantification of the nuclear changes showed that the apoptotic index increased from approximately 3% in untreated cells to approximately 31% following exposure to 500 μM LB (Figure 6B). No statistically significant change was observed at 75 μM, whereas the increase became significant at 150 μM (* *p* < 0.05), was more pronounced at 175 μM (*** *p* < 0.001), and reached the highest level of statistical significance at concentrations ranging from 300 to 500 μM (**** *p* < 0.0001).

Qualitative alterations in cytoskeletal organization were also apparent from 150 μM and became progressively more evident at higher LB concentrations. These changes included cell contraction and rounding, together with a less organized distribution of the F-actin and α-tubulin networks. The most prominent nuclear and cytoskeletal alterations were observed at 400–500 μM. Overall, LB exposure produced a concentration-dependent increase in apoptotic nuclear morphology accompanied by qualitative cytoskeletal reorganization.

### 3.7. LB Increases Caspase-3/7 and Caspase-9 Activity in A375 Cells

**Figure 7:**
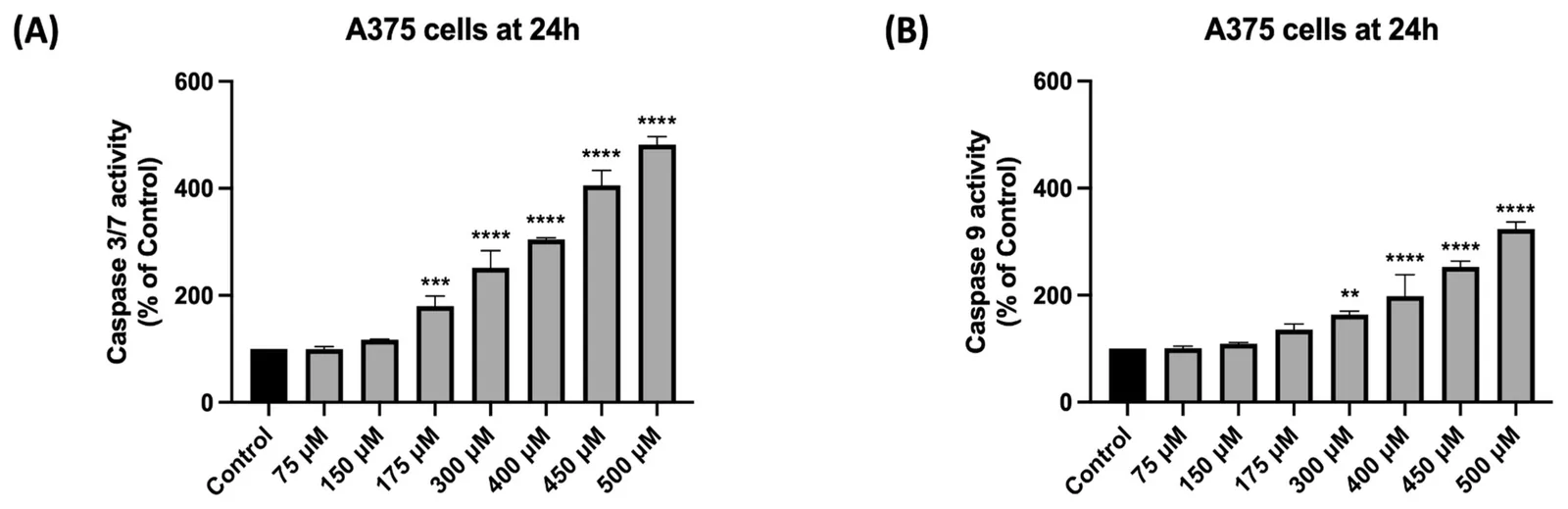
Effects of LB on caspase-3/7 and caspase-9 activity in A375 cells. Cells were exposed to LB at concentrations ranging from 75 to 500 μM for 24 h. (**A**) Caspase-3/7 activity. (**B**) Caspase-9 activity. Luminescence values were normalized to the untreated control, which was assigned a value of 100%. Data are presented as mean ± standard deviation (SD) from three independent experiments, each performed in triplicate. Statistical comparisons between treated and untreated cells were performed using one-way ANOVA followed by Dunnett’s multiple-comparisons test. Statistical significance is indicated as follows: ** *p* < 0.01; *** *p* < 0.001; **** *p* < 0.0001.

To further investigate whether LB’s cytotoxic effects were associated with apoptotic signaling, we evaluated caspase-3/7 and caspase-9 activities in A375 cells after 24 h of treatment. LB induced a concentration-dependent increase in caspase-3/7 activity (Figure 7A). Values measured after exposure to 75 and 150 μM LB remained close to the untreated control, which was assigned a value of 100%, and did not differ significantly from it. At 175 μM, caspase-3/7 activity increased to approximately 180% of the control and reached statistical significance (*** *p* < 0.001). A further increase was observed at concentrations ranging from 300 to 500 μM (**** *p* < 0.0001), with activity reaching approximately 482% of the control at 500 μM.

Caspase-9 activity also increased in a concentration-dependent manner, although the statistically significant response occurred at a higher LB concentration (Figure 7B). Caspase-9 activity did not differ significantly from the control at concentrations ranging from 75 to 175 μM. A significant increase was first observed at 300 μM, where the activity reached approximately 160% of the control (* *p* < 0.01). Caspase-9 activity increased further at 400, 450, and 500 μM (**** *p* < 0.0001), reaching approximately 324% of the control at the highest concentration tested.

Thus, concurrent and statistically significant activation of the initiator caspase-9 and the effector caspases-3/7 was observed at LB concentrations of 300–500 μM. When considered together with the previously observed mitochondrial membrane depolarization and apoptotic nuclear alterations, these findings provide biochemical support for an association between LB-induced cytotoxicity and mitochondria-associated apoptotic signaling in A375 cells.

### 3.8. Qualitative AO/PI Assessment of Labetalol-Induced Cell Death in A375 Cells

**Figure 8:**
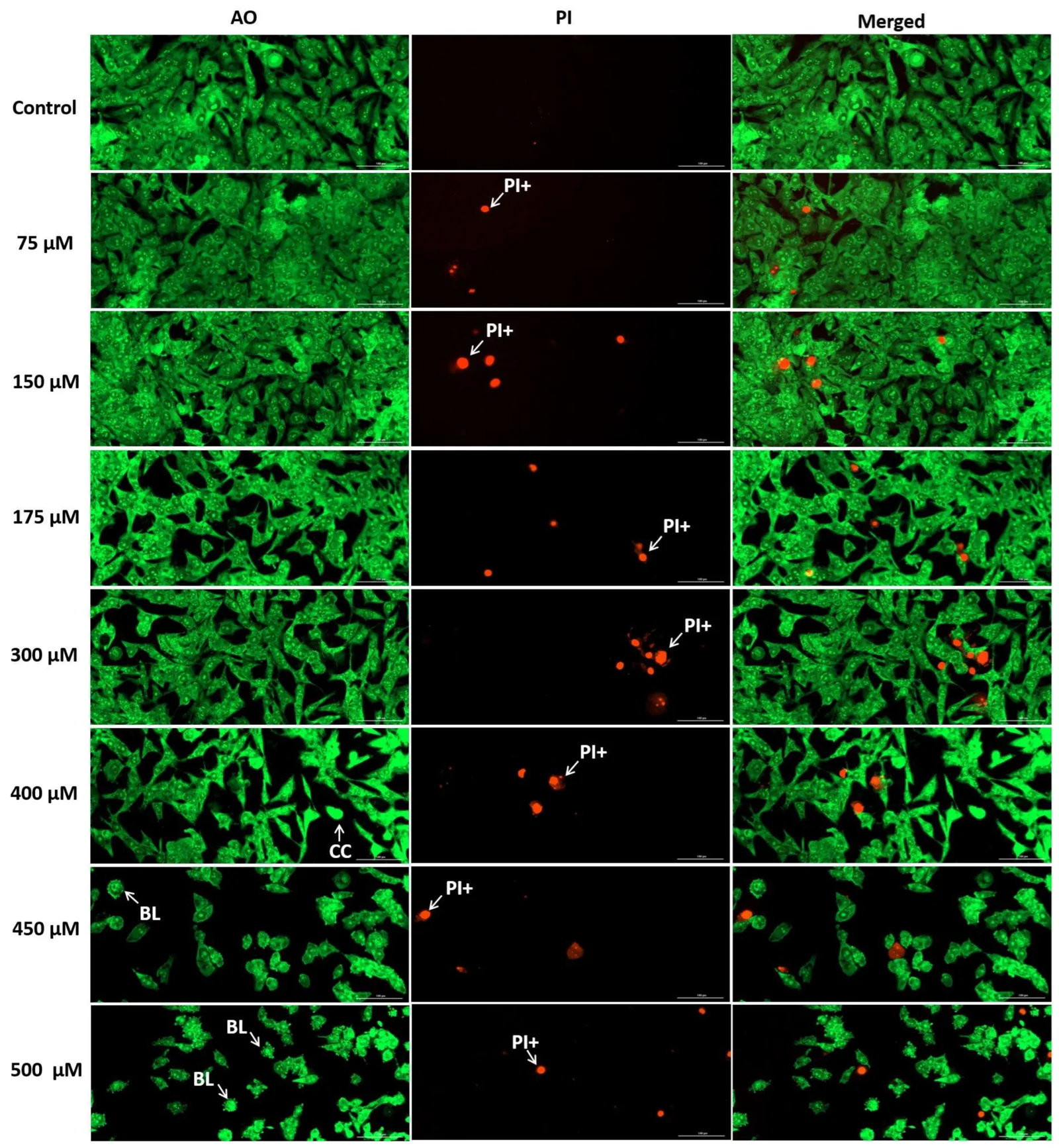
Qualitative AO/PI assessment of LB-induced cell death in A375 cells. Representative fluorescence micrographs of A375 cells following treatment with LB at concentrations ranging from 75 to 500 μM for 24 h. Acridine orange (AO; green channel) stains cellular nucleic acids, whereas propidium iodide (PI; red channel) enters cells with compromised plasma membrane integrity. Merged images illustrate the distribution of PI-negative and PI-positive cells and the morphological changes. Arrows indicate chromatin condensation (CC), membrane blebbing (BL), and PI-positive cells with compromised membrane integrity (PI+). PI positivity alone does not discriminate between late-apoptotic and necrotic cells. All images were acquired using identical acquisition settings. Scale bar: 100 μm.

To further characterize LB-induced changes in cellular morphology and plasma membrane integrity, A375 cells treated with LB for 24 h were examined using AO/PI double staining (Figure 8). Untreated cells predominantly displayed preserved adherent morphology and excluded PI. At concentrations ranging from 75 to 175 μM, most cells remained PI-negative and retained broadly preserved morphology, although occasional PI-positive cells were observed.

At concentrations of 300 μM and above, the representative fields showed more pronounced morphological alterations. These included an apparent reduction in adherent cell density, increased cell rounding and shrinkage, chromatin condensation, and membrane blebbing. PI-positive cells were also observed, indicating loss of plasma membrane integrity. The most marked alterations in cell morphology and adherent cell density were detected following exposure to 450 and 500 μM LB.

Because the AO/PI images were evaluated qualitatively and no stage-specific cell counting was performed, the relative proportions of viable, early-apoptotic, late-apoptotic, and necrotic cells could not be determined. Nevertheless, the occurrence of apoptosis-associated morphological changes together with PI uptake at higher LB concentrations qualitatively supports increased cellular injury and membrane permeabilization. These observations are consistent with the mitochondrial dysfunction and caspase activation detected in the complementary assays.

### 3.9. Evaluation of the Acute Irritation Potential of LB Using the HET-CAM Assay

**Figure 9:**
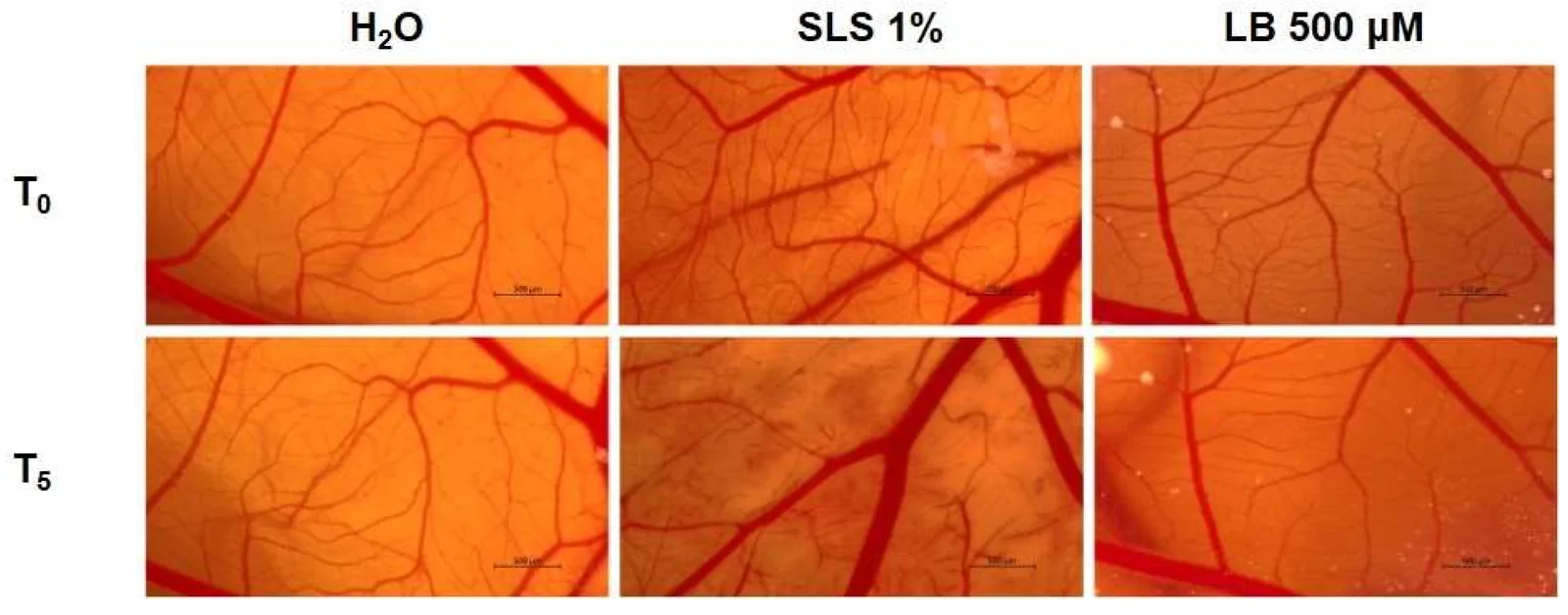
HET-CAM evaluation of the acute irritant potential of LB. Representative stereomicroscope images of the chorioallantoic membranes exposed to distilled water as the negative control, 1% sodium lauryl sulfate (SLS) as the positive control, and 500 μM LB. Images were acquired immediately before sample application (T_0_) and after the 5 min observation period (T_5_). Images are representative of three eggs evaluated per experimental condition. Scale bars represent 500 μm.

The acute irritant potential of LB was evaluated using the HET-CAM assay at 500 μM, corresponding to the highest concentration examined in the cell-based experiments. Distilled water and 1% sodium lauryl sulfate (SLS) were used as negative/vehicle control and positive controls, respectively. The CAM was monitored for 300 s following sample application, and the onset times of hemorrhage, vascular lysis, and coagulation were recorded (Figure 9). For reactions not observed within this interval, an onset time of 301 s was assigned for irritation-score calculation. Each experimental condition was evaluated using three eggs.

The negative/vehicle control did not produce visible vascular alterations during the observation period. Accordingly, an onset time of 301 s was assigned to all three endpoints for irritation-score calculation, resulting in an irritation score of 0.00 ± 0.00. In contrast, 1% SLS rapidly induced hemorrhage, vascular lysis, and coagulation, with mean onset times of 17 ± 2, 22 ± 4, and 15 ± 3 s, respectively (Table 1). The positive control was classified as severely irritant, confirming the expected responsiveness of the assay.

Following exposure to 500 μM LB, no hemorrhage was observed during the 300 s observation period; therefore, an onset time of 301 s was assigned for irritation-score calculation. Very late-onset vascular lysis and coagulation were recorded at 278 ± 5 and 295 ± 5 s, respectively, while the overall vascular architecture remained largely preserved in the representative images. These changes resulted in a mean irritation score of approximately 0.71 ± 0.27, which remained within the non-irritant range according to the applied classification criteria.

## 4. Discussion

The present study provides an initial integrated evaluation of labetalol (LB) as a candidate for melanoma-directed drug repurposing. For this purpose, A375 melanoma cells and HaCaT keratinocytes were used as in vitro cellular models.

A375 cells were selected as the melanoma model because they represent a well-established and widely used in vitro model in melanoma research. Their relevance is further supported by their origin from a primary human melanoma and their well-characterized molecular background, including the BRAF^V600E mutation and alterations in CDKN2A, involving molecular pathways highly relevant to melanoma development and progression [34].

HaCaT cells are frequently employed as non-malignant skin-derived comparators in preclinical antimelanoma studies, facilitating comparison with previously reported findings [35,36,37]. Their selection was also supported by the close physiological relationship between melanocytes and keratinocytes within the epidermis, where a single melanocyte interacts with approximately 30–40 surrounding keratinocytes [38]. From an experimental perspective, HaCaT cells offer a stable and reproducible proliferative model and can be maintained under conditions more comparable to those used for melanoma cells. By contrast, primary melanocytes generally require specialized culture media containing growth factors and other components that may influence signaling responses [39,40,41].

Across complementary assays, LB reduced A375 melanoma cell viability and induced morphological, lysosomal, mitochondrial, cytoskeletal, and apoptotic changes after 24 h of exposure. The response was concentration-dependent and became most pronounced at high micromolar concentrations. In parallel experiments, HaCaT immortalized keratinocytes were considerably less affected, while the LB-containing solution produced a HET-CAM irritation score within the non-irritant range. However, the relatively high effective concentrations, limited cellular comparisons, and lack of receptor-specific experiments require cautious interpretation of LB’s therapeutic and translational relevance.

The IC_50_ of 422.5 µM in A375 cells indicates that LB has modest potency under the present experimental conditions. In HaCaT cells, an IC_50_ was not reached within the investigated concentration range, and approximately 89% viability was maintained at 500 µM. These findings indicate distinct descriptive response profiles under the tested conditions. Direct experimental evidence regarding the anticancer activity of LB remains scarce. In HepG2 hepatocellular carcinoma cells, LB reduced viability at relatively high concentrations, although the proposed interaction with cell division cycle 20 (CDC20) was supported mainly by computational analyses [42]. LB also reduced the viability of three-dimensional uveal melanoma spheroids at concentrations of at least 150 µM after prolonged exposure [20]. These observations are directionally consistent with the present findings but cannot be used for a quantitative comparison because of differences in tumor origin, culture dimensionality, exposure time, and assay endpoints. In contrast, propranolol and carvedilol have generally shown activity at lower concentrations in melanoma models. Propranolol reduced A375 and primary acral melanoma cell viability, with A375 IC_50_ values of approximately 65–98 µM depending on exposure duration, while carvedilol was more potent than propranolol in a recent comparative analysis [15,16]. Thus, the antitumor activity of adrenergic antagonists is not uniform across the pharmacological class, and the lower apparent potency of LB may reflect differences in receptor profile, physicochemical properties, intracellular accumulation, or receptor-independent effects.

The concentration-related morphological changes in A375 cells were consistent with the viability findings. Cell rounding, reduced spreading, shrinkage, detachment, and lower adherent cell density became increasingly apparent within the concentration range producing the greatest reduction in MTT signal, whereas comparable damage was not evident in HaCaT cultures. Shrinkage and the formation of membrane-bound fragments are classical morphological features of apoptosis [43], but bright-field morphology alone cannot discriminate apoptosis from necrosis, mitotic rounding, impaired adhesion, or reversible cellular stress. Previous studies have similarly shown that propranolol can inhibit proliferation and induce apoptotic morphology in melanoma cells, including A375 cells and cultures derived from primary and metastatic melanomas [15,44]. Therefore, the morphological observations are consistent with the concentration-dependent A375 response detected by MTT and with the limited morphological changes observed in HaCaT cells.

NRU provided a complementary measure of the A375 response because it depends on the capacity of viable cells to retain the dye within acidic lysosomal compartments. The overall concentration–response pattern closely paralleled that obtained using MTT, and the two assays produced almost identical residual signals at 500 µM. This convergence strengthens the evidence for a substantial reduction in the viable cell population at the highest concentration. The thresholds of statistical significance nevertheless differed: the MTT response was significant from 75 µM, whereas the NRU decrease became significant at 175 µM. This difference should not be interpreted as evidence that NRU was more sensitive to LB, since statistical significance also depends on experimental variability and the dynamic range of each assay. Because neutral-red retention depends on lysosomal acidification and membrane integrity as well as on the number of viable adherent cells, the present data cannot distinguish direct lysosomal impairment from a general reduction in viable cell number [45]. Comparative studies have shown that NRU and MTT frequently yield concordant cytotoxicity profiles but may differ in sensitivity according to the compound, cell type, exposure duration, and mechanism of injury [46,47]. Moreover, propranolol has been reported to alter lysosomal dye accumulation and drug sequestration in vascular sarcoma cells through mechanisms largely independent of β-adrenergic receptor blockade [48]. This observation provides contextual support for a possible interaction between some β-blockers and the lysosomal compartment, but the current NRU findings do not demonstrate that lysosomes are a direct or primary target of LB.

The additional vehicle-only experiment also provides context for interpretation of the viability findings. Across the exact DMSO concentration range present in the LB treatment solutions (0.075–0.50% *v*/*v*), no statistically significant reduction in MTT-derived viability was observed in either A375 or HaCaT cells. Moreover, the DMSO-only concentration series did not reproduce the concentration-dependent viability loss observed with LB. These findings indicate that vehicle cytotoxicity alone is unlikely to explain the principal MTT response. Nevertheless, because this additional evaluation was limited to MTT-derived viability, it does not exclude possible endpoint-specific effects of DMSO on mitochondrial, lysosomal, cytoskeletal, or apoptotic readouts. Accordingly, the mechanistic findings should be interpreted as associations requiring confirmation under fully vehicle-matched experimental conditions.

Mitochondrial dysfunction emerged as a prominent component of the A375 response. JC-1 analysis demonstrated a concentration-dependent reduction in mitochondrial membrane potential, with a significant decrease beginning at 150 µM and a pronounced loss of the aggregate/monomer ratio at 300–500 µM. Because JC-1 accumulation and aggregate formation depend on the electrochemical gradient across the inner mitochondrial membrane, the observed ratio change supports mitochondrial depolarization rather than directly quantifying viable cell number [49,50]. The overlap between the concentration ranges associated with mitochondrial depolarization, reduced metabolic activity, impaired NRU, and subsequent apoptotic changes indicates that mitochondrial injury is closely associated with LB-induced cytotoxicity. A similar response was reported in H9c2 rat cardiomyoblasts, in which a cytotoxic concentration of LB caused mitochondrial depolarization, ATP depletion, an increased Bax/Bcl-2 ratio, and activation of caspase-9 and caspase-3 [51]. Mitochondrial depolarization and intrinsic apoptotic signaling have also been described following propranolol treatment in A375 melanoma cells and in multiple myeloma cells [15,52]. Although these studies support the biological plausibility of the present findings, the different cellular and pharmacological contexts prevent direct extrapolation. Furthermore, because all endpoints were assessed after the same 24 h exposure, the results do not establish whether mitochondrial depolarization precedes or causes the reduction in viability.

The MitoTracker Red CMXRos images provided complementary qualitative evidence of altered mitochondrial organization, particularly at the higher LB concentrations, where the fluorescence pattern changed in parallel with cell contraction, rounding, and reduced cell density. Interpretation of these images requires caution because CMXRos is a lipophilic cationic probe whose mitochondrial accumulation depends substantially on membrane potential [53]. Pronounced depolarization may therefore reduce or redistribute the probe and compromise the reliability of mitochondrial segmentation and morphometric interpretation [54]. In addition, mitochondrial morphology in melanoma is strongly influenced by the cellular signaling and metabolic state. BRAF^V600E-associated mitogen-activated protein kinase signaling promotes DRP1-dependent mitochondrial division in A375 cells, whereas pathway inhibition can induce extensive fusion [55]. Conversely, mitochondrial shortening, fragmentation, and clustering have been associated with mitochondrial depolarization, caspase activation, and apoptotic nuclear changes in A375 cells exposed to apoptosis-inducing agents [45]. Mitochondrial structural alterations have also been reported in cancer cells exposed to propranolol under metabolically stressful conditions [56]. Accordingly, the current images support an association between LB-induced injury and altered mitochondrial distribution but do not independently demonstrate fragmentation, fusion, fission, aggregation, or changes in mitochondrial mass. Such statements would require higher-resolution imaging and quantitative assessment of mitochondrial length, branching, aspect ratio, form factor, and network connectivity [57].

Nuclear and cytoskeletal imaging further supported the development of an apoptotic phenotype. The apoptotic index increased progressively from approximately 3% in untreated cultures to approximately 31% at 500 µM, with a statistically significant increase from 150 µM. This pattern indicates a gradual transition from limited cellular stress at lower concentrations to overt nuclear injury as LB exposure increased. The observed chromatin condensation, nuclear shrinkage, and fragmentation are compatible with apoptosis, although morphology-based scoring cannot independently establish a specific death pathway. Similar nuclear changes have been reported in propranolol-treated A375 cells, together with biochemical activation of intrinsic apoptosis [15]. In the same cell line, apoptotic nuclear alterations have also been associated with actin reorganization and marked changes in cellular morphology following other stimuli [58]. These comparisons support the interpretation of the LB-induced nuclear phenotype without implying that the drugs or stimuli act through identical molecular mechanisms.

The concomitant changes in F-actin and α-tubulin organization can be interpreted as apoptosis-associated cytoskeletal remodeling rather than as evidence of direct cytoskeletal targeting by LB. During apoptosis, the interphase microtubule network may initially disassemble and subsequently reorganize into non-centrosomal arrays involved in chromatin redistribution and apoptotic-body formation [59]. Nevertheless, cytoskeletal disorganization is not specific to apoptosis. In HT-144 melanoma cells, cinnamic acid-induced apoptosis and microtubule alterations were shown to be at least partially independent, while propranolol modified actin dynamics and migration in osteosarcoma cells under predominantly cytostatic conditions [60,61]. Thus, the altered F-actin and α-tubulin patterns cannot establish filament depolymerization, changes in protein abundance, or a causal relationship with apoptosis. Their concurrence with the concentration-dependent increase in apoptotic nuclei and mitochondrial depolarization nevertheless strengthens the evidence that substantial cellular remodeling accompanies the cytotoxic response to LB.

The caspase results provide biochemical support for this interpretation. Caspase-3/7 activity increased significantly from 175 µM and reached approximately 482% of the control value at 500 µM, whereas caspase-9 activation became significant at 300 µM and reached approximately 324% of control. The concurrent activation of the initiator caspase-9 and effector caspases-3/7 at 300–500 µM coincided with pronounced mitochondrial depolarization and nuclear alterations. During canonical intrinsic apoptosis, mitochondrial outer membrane permeabilization promotes cytochrome c release and apoptosome-dependent activation of caspase-9, which subsequently activates downstream effector caspases [62]. However, the concurrent mitochondrial depolarization and caspase activation observed in the present study support an association with mitochondria-associated apoptotic signaling rather than establishing a causal intrinsic apoptotic cascade. Because these endpoints were assessed at a single time point following 24 h of LB exposure, their temporal sequence cannot be determined, and mitochondrial depolarization cannot be concluded to precede or directly cause caspase activation. Nevertheless, caspase-9, caspase-3, and caspase-7 have distinct and partly non-redundant roles, and the combined Caspase-Glo 3/7 signal cannot distinguish the individual contributions of the two effector caspases [63]. The lower concentration threshold observed for caspase-3/7 than for caspase-9 does not exclude mitochondrial signaling because the assays differ in substrates, background, analytical range, and potentially in the kinetics of the measured enzymes. At the same time, it prevents the conclusion that caspase-9 was the only initiator involved. Death-receptor signaling or cross-talk between intrinsic and extrinsic pathways may also contribute to effector-caspase activation.

The caspase profile is consistent with previous evidence regarding adrenergic antagonists. In propranolol-treated A375 cells, increased Bax expression, decreased Bcl-2 expression, cytochrome c release, and cleavage of caspase-9 and caspase-3 supported activation of the intrinsic pathway [15]. In uveal melanoma models, nebivolol reduced viability and cellular ATP while increasing caspase-3/7 activity, demonstrating that metabolic stress and apoptotic execution may also occur with other β-blockers [64]. The findings obtained in LB-treated cardiomyoblasts are particularly relevant to mechanistic plausibility because mitochondrial depolarization and activation of caspase-9 and caspase-3 occurred without a significant increase in cleaved caspase-8 [51]. Nevertheless, that study addressed cardiotoxicity in a non-malignant cardiac model and therefore cannot establish melanoma-specific activity. In addition, the absence of cytochrome c measurements, caspase-8 assessment, temporal profiling, and inhibitor-based rescue experiments in the present study limits further mechanistic attribution of the observed apoptotic response.

AO/PI staining added qualitative morphological evidence of LB-induced cell death. Up to 175 µM, most examined cells remained PI-negative and retained comparatively preserved morphology. At 300 µM and above, lower adherent cell density, rounding, shrinkage, chromatin condensation, and membrane blebbing became increasingly apparent, while PI-positive cells were more evident at 450–500 µM. Because acridine orange permeates cells irrespective of membrane integrity, green fluorescence alone does not confirm viability when apoptotic nuclear alterations are present. Similarly, PI uptake demonstrates plasma membrane permeabilization but cannot independently distinguish late apoptosis from primary necrosis [65,66]. Therefore, PI-negative cells showing chromatin condensation or blebbing are compatible with early apoptosis, whereas PI-positive cells may represent late-apoptotic, secondarily necrotic, or primarily necrotic populations. Comparable AO/PI transitions, accompanied by chromatin condensation and nuclear fragmentation, have been described in A375 cells exposed to other apoptosis-inducing compounds [67]. Pro-apoptotic effects have also been reported for propranolol in cutaneous and uveal melanoma models [68]. Although these comparisons provide relevant biological context, the qualitative and single-time-point nature of the present AO/PI evaluation prevents stage-specific quantification or confirmation of progression from apoptosis to secondary necrosis.

When considered collectively, the findings from nuclear morphology, cytoskeletal organization, mitochondrial membrane potential, caspase activation, and AO/PI staining are more informative than any individual endpoint. Their concentration-dependent convergence supports apoptosis as a major component of LB-induced A375 cell death and implicates mitochondria-associated caspase signaling in this response. At the same time, the data do not define a complete causal sequence. Mitochondrial depolarization may represent an initiating event, a consequence of apoptotic commitment, or part of a bidirectional amplification process. Likewise, cytoskeletal remodeling may contribute to cell detachment and apoptotic execution without representing a primary molecular target. Importantly, the experiments did not determine whether these effects resulted from β-adrenergic or α_1_-adrenergic receptor antagonism. The high micromolar concentrations required for substantial activity also leave open the possibility of receptor-independent cellular effects. These distinctions are important for a drug-repurposing strategy because confirmation of anticancer activity alone is insufficient without defining the mechanism, achievable exposure, and therapeutic margin.

The HET-CAM experiment provided a preliminary evaluation of acute irritation associated with the LB-containing test solution. At 500 µM, the mean irritation score of 0.71 ± 0.27 remained within the non-irritant range of the applied classification system. No hemorrhage was recorded during the 300 s observation period, and vascular lysis and coagulation appeared only near its end. In contrast, 1% SLS rapidly produced all three reactions and a high irritation score, confirming the responsiveness of the assay. Because the HET-CAM scoring system assigns greater weight to early vascular reactions, the late onset of lysis and coagulation contributed only modestly to the final score; these changes should nevertheless be acknowledged rather than described as absent. The findings therefore indicate low acute irritation potential under the tested conditions, rather than a complete absence of vascular effects.

HET-CAM was developed as a rapid alternative method for screening hemorrhage, vascular lysis, and coagulation on the CAM [32]. Subsequent evaluations have supported its value for identifying substances with low acute irritation potential while emphasizing the effects of protocol selection, scoring criteria, and reference controls [69,70,71]. Concordance between non-irritant HET-CAM classifications and acceptable tolerability in product-use studies further supports its role as an initial screening tool [72,73]. Cell-based testing and HET-CAM have also been used together during the preclinical evaluation of topical delivery systems, providing complementary information regarding cellular compatibility and acute vascular irritation [74]. However, irritation may vary substantially with formulation composition, as demonstrated by formulation-dependent HET-CAM classifications for brimonidine-containing nanoemulsions [33]. This consideration is relevant because the present experiment evaluated an unformulated LB solution rather than a finalized pharmaceutical product. Excipients, pH, osmolarity, drug release, and tissue exposure may alter the irritation profile of a future formulation.

The HET-CAM result and the relative preservation of HaCaT metabolic activity address different aspects of non-malignant tissue response. HaCaT testing evaluated cellular metabolic activity after 24 h, whereas HET-CAM monitored immediate vascular events for only 5 min. Their apparent agreement therefore represents complementary preliminary evidence rather than confirmation of dermal safety. Nevertheless, the combination of concentration-dependent activity in A375 cells, mechanistically coherent evidence of mitochondria-associated apoptosis, limited HaCaT effects, and low acute HET-CAM irritation provides a reasonable basis for continued evaluation of LB within a melanoma-directed repurposing strategy. The principal challenge is that substantial antitumor effects occurred at relatively high concentrations. Establishing whether adequate local exposure can be achieved without injury to normal cutaneous tissues, and whether the response extends beyond A375 cells, will be essential for determining the translational potential of LB.

### Study Limitations and Future Directions

Several limitations of the present study should be acknowledged.

The use of a single human melanoma cell line (A375) represents a limitation of the present study, as it does not capture the molecular and phenotypic heterogeneity of cutaneous melanoma. Consequently, the findings cannot be generalized across melanoma models and should be validated in additional cell lines with distinct molecular backgrounds. In addition, HaCaT cells, although useful as a non-tumoral skin-derived comparator, are spontaneously immortalized and do not fully reproduce the biology of primary human keratinocytes or intact normal skin [75]. The lack of primary human melanocytes as an additional non-malignant comparator further limits the assessment of melanoma-specific selectivity.

Most experiments were performed after a single 24 h exposure, and the most pronounced cytotoxic effects occurred at relatively high micromolar LB concentrations. Whether comparable concentrations can be achieved and maintained in melanoma tissue, particularly through local administration, remains unknown. In addition, MTT and NRU are indirect viability-related endpoints and do not establish cell number or irreversible loss of proliferative capacity. A further limitation is the absence of assay-specific positive controls for the mechanistic endpoints. Although concentration-dependent changes were consistently observed across complementary assays, the lack of dedicated positive controls limits the independent validation of assay performance and pathway-specific responses. An additional limitation of the original experimental design is the absence of a constant vehicle-matched control across all LB concentrations. To further assess the potential contribution of the vehicle, DMSO-only exposure at the exact concentrations corresponding to the LB treatment groups (0.075–0.50% *v*/*v*) was subsequently evaluated using the MTT assay in both A375 and HaCaT cells. No statistically significant reduction in metabolic viability was observed across this concentration range, and the DMSO-only profile did not reproduce the concentration-dependent loss of viability observed following LB exposure. These additional findings indicate that DMSO alone was unlikely to account for the principal MTT viability response. Nevertheless, because the additional vehicle evaluation was limited to MTT-derived viability, it does not exclude possible endpoint-specific effects of DMSO or compound–vehicle interactions in the mechanistic assays. Therefore, confirmation of the mechanistic findings using an identical final vehicle concentration across all treatment conditions remains warranted in future studies.

Future investigations should therefore validate the response in additional melanoma cell lines with different molecular backgrounds and in primary keratinocytes and melanocytes, followed by three-dimensional melanoma and reconstructed-skin models. Time-course, recovery, and clonogenic experiments, together with appropriate assay-specific positive controls, quantitative Annexin V/PI analysis, cytochrome c release, Bax/Bcl-2 and cleaved-caspase assessment, and caspase-inhibition studies, would clarify and strengthen the interpretation of the apoptotic response. Receptor-expression studies and comparisons with selective antagonists, agonists, or genetic modulation are also needed to distinguish receptor-mediated from receptor-independent activity. For local repurposing, evaluation of a finalized formulation should include multiple LB concentrations, an appropriate vehicle control, skin permeation and retention, reconstructed epidermis or ex vivo skin testing, repeated exposure, and subsequent in vivo assessment. Despite these limitations, the convergence of complementary findings supports continued evaluation of LB as a melanoma-directed repurposing candidate, while these studies will be essential to establish reproducibility, mechanism, safety, achievable exposure, and translational relevance.

## 5. Conclusions

The present study provides preliminary preclinical evidence supporting the investigation of labetalol within a melanoma-directed drug-repurposing strategy. LB reduced A375 melanoma cell viability in a concentration-dependent manner after 24 h of exposure, with the most pronounced effects occurring at concentrations ranging from 400 to 500 μM. Under the same experimental conditions, HaCaT immortalized keratinocytes maintained approximately 89% viability at the highest concentration tested. However, these findings do not establish general tumor selectivity or a therapeutic safety margin. The complementary experimental findings indicate that the reduction in A375 cell viability was accompanied by impaired NRU, loss of mitochondrial membrane potential, alterations in mitochondrial staining distribution, apoptotic nuclear morphology, cytoskeletal reorganization, and increased caspase-9 and caspase-3/7 activities. Together with the qualitative AO/PI observations, these results support an association between LB-induced cellular injury and mitochondria-associated apoptotic signaling. However, the present data do not establish the complete causal sequence of cell death or determine whether the observed effects depend on α_1_- or β-adrenergic receptor antagonism. At 500 μM, the LB-containing test solution produced a mean HET-CAM irritation score within the non-irritant range, providing preliminary evidence of low acute irritation potential under the conditions employed. This result should not be interpreted as confirmation of dermal safety or of the tolerability of a future topical formulation.

Overall, the convergence of metabolic, mitochondrial, morphological, cytoskeletal, and biochemical findings identifies LB as a candidate for further preclinical evaluation rather than as an established anti-melanoma therapy. Studies in additional cutaneous melanoma models, primary non-tumoral cells, three-dimensional cultures, and appropriate in vivo systems are needed to confirm the reproducibility and specificity of the response. Receptor-focused experiments, pharmacokinetic and skin-permeation studies, and evaluation of a finalized pharmaceutical formulation will be essential for determining whether the observed in vitro activity can be translated into a viable repurposing strategy.

## Figures and Tables

**Table 1:** HET-CAM response parameters following exposure to distilled water, 1% SLS, and 500 μM LB. The onset times of hemorrhage (t_H_), vascular lysis (t_L_), and coagulation (t_C_), together with the calculated irritation scores (IS), are presented as mean ± SD (*n* = 3). Irritation was classified according to the following IS ranges: 0–0.9, non-irritant; 1–4.9, slightly irritant; 5–8.9, moderately irritant; and 9–21, severely irritant.

	H_2_O	1% SLS	LB 500 μM
IS	0.00 ± 0.00	17 ± 2	0.71 ± 0.27
t_H_ (s)	301 ± 0	17 ± 2	301 ± 0
t_L_ (s)	301 ± 0	22 ± 4	278 ± 5
t_C_ (s)	301 ± 0	15 ± 3	295 ± 5

## Data Availability

All the data obtained are included in the present article. Further inquiries can be directed to the corresponding author.

## Abbreviations

The following abbreviations are used in this manuscript:

AKT	Protein kinase B
AO	Acridine orange
ANOVA	Analysis of variance
ATP	Adenosine triphosphate
ATCC	American Type Culture Collection
Bax	Bcl-2-associated X protein
Bcl-2	B-cell lymphoma 2
BL	Membrane blebbing
BRAF	B-Raf proto-oncogene, serine/threonine kinase
BSA	Bovine serum albumin
CAM	Chorioallantoic membrane
CC	Chromatin condensation
CDC20	Cell division cycle 20
CLS	Cell Lines Service
DMEM	Dulbecco’s Modified Eagle Medium
DMSO	Dimethyl sulfoxide
DRP1	Dynamin-related protein 1
F-actin	Filamentous actin
FBS	Fetal bovine serum
HET-CAM	Hen’s Egg Test–Chorioallantoic Membrane
IC_50_	Half-maximal inhibitory concentration
IgG	Immunoglobulin G
IS	Irritation score
JC-1	5,5′,6,6′-tetrachloro-1,1′,3,3′-tetraethylbenzimidazolylcarbocyanine iodide
LB	labetalol
MAPK	Mitogen-activated protein kinase
MEK	Mitogen-activated protein kinase kinase
MTT	3-(4,5-dimethylthiazol-2-yl)-2,5-diphenyltetrazolium bromide
NRU	Neutral red uptake
PBS	Phosphate-buffered saline
PI	Propidium iodide
SD	Standard deviation
SLS	Sodium lauryl sulfate
ΔΨm	Mitochondrial membrane potential

## References

[1] Țăpoi D.A., Derewicz D., Gheorghișan-Gălățeanu A.-A., Dumitru A.V., Ciongariu A.M., Costache M. (2023). The Impact of Clinical and Histopathological Factors on Disease Progression and Survival in Thick Cutaneous Melanomas. Biomedicines.

[2] Țăpoi D.A., Gheorghișan-Gălățeanu A.-A., Gosman L.M., Derewicz D., Costache M. (2024). The Prognostic Value of Proliferative Activity in Cutaneous Melanoma: A Pilot Study Evaluating the Mitotic Rate and Ki67 Index to Predict Patient Outcomes. Biomedicines.

[3] Scampa M., Mégevand V., Viscardi J.A., Giordano S., Kalbermatten D.F., Oranges C.M. (2022). Melanoma of the Scalp and Neck: A Population-Based Analysis of Survival and Treatment Patterns. Cancers.

[4] De Giorgi V., Geppetti P., Lupi C., Benemei S. (2020). The Role of β-Blockers in Melanoma. J. Neuroimmune Pharmacol..

[5] Ionita I., Malita D., Dehelean C., Olteanu E., Marcovici I., Geamantan A., Chiriac S., Roman A., Radu D. (2023). Experimental Models for Rare Melanoma Research—The Niche That Needs to Be Addressed. Bioengineering.

[6] Garutti M., Bonin S., Buriolla S., Bertoli E., Pizzichetta M.A., Zalaudek I., Puglisi F. (2021). Find the Flame: Predictive Biomarkers for Immunotherapy in Melanoma. Cancers.

[7] Olbryt M., Rajczykowski M., Widłak W. (2020). Biological Factors behind Melanoma Response to Immune Checkpoint Inhibitors. Int. J. Mol. Sci..

[8] Indini A., Massi D., Pirro M., Roila F., Grossi F., Sahebkar A., Glodde N., Bald T., Mandalà M. (2022). Targeting Inflamed and Non-Inflamed Melanomas: Biological Background and Clinical Challenges. Semin. Cancer Biol..

[9] Song L., Yang Y., Tian X. (2024). Current Knowledge about Immunotherapy Resistance for Melanoma and Potential Predictive and Prognostic Biomarkers. Cancer Drug Resist..

[10] Armando R.G., Mengual Gómez D.L., Gomez D.E. (2020). New Drugs Are Not Enough—Drug Repositioning in Oncology: An Update. Int. J. Oncol..

[11] Cortés H., Reyes-Hernández O.D., Alcalá-Alcalá S., Bernal-Chávez S.A., Caballero-Florán I.H., González-Torres M., Sharifi-Rad J., González-Del Carmen M., Figueroa-González G., Leyva-Gómez G. (2021). Repurposing of Drug Candidates for Treatment of Skin Cancer. Front. Oncol..

[12] Spitschak A., Gupta S., Singh K.P., Logotheti S., Pützer B.M. (2023). Drug Repurposing at the Interface of Melanoma Immunotherapy and Autoimmune Disease. Pharmaceutics.

[13] Qiao G., Chen M., Bucsek M.J., Repasky E.A., Hylander B.L. (2018). Adrenergic Signaling: A Targetable Checkpoint Limiting Development of the Antitumor Immune Response. Front. Immunol..

[14] Dal Monte M., Calvani M., Cammalleri M., Favre C., Filippi L., Bagnoli P. (2019). β-Adrenoceptors as Drug Targets in Melanoma: Novel Preclinical Evidence for a Role of β3-Adrenoceptors. Br. J. Pharmacol..

[15] Zhou C., Chen X., Zeng W., Peng C., Huang G., Li X., Ouyang Z., Luo Y., Xu X., Xu B. (2016). Propranolol induced G0/G1/S phase arrest and apoptosis in melanoma cells via AKT/MAPK pathway. Oncotarget.

[16] Rama L., Almeida M., Jose J., Pereira M.L., Oliveira M. (2025). Beta-Blockers as Potential Adjuvants in Melanoma Treatment. Toxics.

[17] McCourt C., Coleman H.G., Murray L.J., Cantwell M.M., Dolan O.M., Powe D.G., Cardwell C.R. (2014). Beta-Blocker Usage after Malignant Melanoma Diagnosis and Survival: A Population-Based Nested Case-Control Study. Br. J. Dermatol..

[18] Khan Z., Demirtaş E., Kıroğlu O., Karataş Y. (2022). Beta-Adrenergic Blockers’ Supportive and Adverse Role in Hypertension: A Review of Three Generations. Pak. J. Med. Dent..

[19] Cleveland K.H., Liang S., Chang A., Huang K.M., Chen S., Guo L., Huang Y., Andresen B.T. (2019). Carvedilol Inhibits EGF-Mediated JB6 P+ Colony Formation through a Mechanism Independent of Adrenoceptors. PLoS ONE.

[20] Farhoumand L.S., Fiorentzis M., Kraemer M.M., Sak A., Stuschke M., Rassaf T., Hendgen-Cotta U.B., Bechrakis N.E., Berchner-Pfannschmidt U. (2022). The Adrenergic Receptor Antagonist Carvedilol Elicits Anti-Tumor Responses in Uveal Melanoma 3D Tumor Spheroids and May Serve as Co-Adjuvant Therapy with Radiation. Cancers.

[21] Ilieva Y., Dimitrova L., Zaharieva M.M., Kaleva M., Alov P., Tsakovska I., Pencheva T., Pencheva-El Tibi I., Najdenski H., Pajeva I. (2021). Cytotoxicity and Microbicidal Activity of Commonly Used Organic Solvents: A Comparative Study and Application to a Standardized Extract from Vaccinium macrocarpon. Toxics.

[22] Buchser W., Collins M., Garyantes T., Guha R., Haney S., Lemmon V., Li Z., Trask O.J., Markossian S., Grossman A., Baskir H., Brimacombe K., Hall M.D. (2012). Assay Development Guidelines for Image-Based High Content Screening, High Content Analysis and High Content Imaging. Assay Guidance Manual.

[23] Piras F., Sogos V., Camola A., Pollastro F., Rosa A. (2026). The Natural Phloroglucinol α-Pyrone Arzanol Protects HaCaT Keratinocytes from Lipopolysaccharide and Polyriboinosinic-Polyribocytidylic Acid-Induced Damage and Promotes Reparative Mechanisms. Appl. Sci..

[24] Yu L., Tian Y., Gao A., Shi Z., Liu Y., Li C. (2015). Bi-Module Sensing Device to In Situ Quantitatively Detect Hydrogen Peroxide Released from Migrating Tumor Cells. PLoS ONE.

[25] Vicaș L.G., Marian N.A., Ali D.H., Duteanu N., Svera P., Dehelean C., Vlase A.-M., Frenț O.-D., Dejeu I.-L., Negrean R.A. (2026). Potentiation of the Pharmacological Effects of an Aristolochia clematitis L. Extract by Loading into Liposomes Facilitating Release to HaCaT Cells. Pharmaceutics.

[26] Repetto G., del Peso A., Zurita J.L. (2008). Neutral red uptake assay for the estimation of cell viability/cytotoxicity. Nat. Protoc..

[27] Talpos S., Chioran D., Alexandru G.C., Dinu Ș., Coricovac E.-D., Smeu A., Ali D.H., Szuhanek C., Popa M. (2025). Silibinin Triggers Mitochondrial Apoptosis and Declines Clonogenic Potential in Detroit 562 Human Pharyngeal Carcinoma Cells. Medicina.

[28] Smeu A., Minda D., Boru C., Vlaia L., Vlaia V., Dehelean C.A., Liga S., Puenea G., Muntean D.L. (2025). Betulinic Acid-Loaded Oleogel as a Novel Pharmaceutical Formulation for Potential Cutaneous Applications: Development, Characterization, and Biosafety Profile. Life.

[29] Marcovici I., Chioibas R., Zupko I., Pinzaru I., Moaca A., Ledeti A., Barbu-Tudoran L., Geamantan A., Predescu I., Dehelean C.A. (2025). Preclinical pharmaco-toxicological screening of biomimetic melanin-like nanoparticles as a potential therapeutic strategy for cutaneous melanoma. Front. Pharmacol..

[30] Feher A., Căta A., Haj Ali D., Bora L., Magyari-Pavel I.Z., Vlase A.-M., Avram Ș., Vlase L., Ungureanu D., Dinu Ș. (2026). Phytochemical Characterization and Biological Assessment of Geranium robertianum L. Ethanolic Extract on Human Salivary Gland Carcinoma Cells. Antioxidants.

[31] Kis A.M., Macasoi I., Paul C., Radulescu M., Buzatu R., Watz C.G., Cheveresan A., Berceanu D., Pinzaru I., Dinu S. (2022). Methotrexate and Cetuximab—Biological Impact on Non-Tumorigenic Models: In Vitro and In Ovo Assessments. Medicina.

[32] Luepke N.P. (1985). Hen’s egg chorioallantoic membrane test for irritation potential. Food Chem. Toxicol..

[33] Smail S.S. (2024). Ex Vivo Irritation Evaluation of a Novel Brimonidine Nanoemulsion Using the Hen’s Egg Test on Chorioallantoic Membrane (HET-CAM). Cureus.

[34] Avram S., Coricovac D.-E., Pavel I.Z., Pinzaru I., Ghiulai R., Baderca F., Soica C., Muntean D., Branisteanu D.E., Spandidos D.A. (2017). Standardization of A375 Human Melanoma Models on Chicken Embryo Chorioallantoic Membrane and Balb/c Nude Mice. Oncol. Rep..

[35] Liga S., Vodă R., Lupa L., Pop R., Haj Ali D., Predescu I.-A., Dehelean C.A., Péter F. (2026). Comparative In Vitro Response of HaCaT and A375 Melanoma Cells to Zinc Oxide Nanoparticles Synthesized Using Puerarin. J. Xenobiotics.

[36] Rosa A., Piras F., Pollastro F., Sogos V., Appendino G., Nieddu M. (2024). Comparative Evaluation of Anticancer Activity of Natural Methoxylated Flavones Xanthomicrol and Eupatilin in A375 Skin Melanoma Cells. Life.

[37] Ndlovu B., De Kock M., Klaasen J., Rahiman F. (2021). In Vitro Comparison of the Anti-Proliferative Effects of *Galenia africana* on Human Skin Cell Lines. Sci. Pharm..

[38] Nordlund J.J. (2007). The Melanocyte and the Epidermal Melanin Unit: An Expanded Concept. Dermatol. Clin..

[39] Jiménez-González V., Benítez G., Pastor J.E., López-Lázaro M., Calderón-Montaño J.M. (2026). Screening for Selective Anticancer Activity of Extracts from 59 Plant Species Collected in Southern Spain (Andalusia). Pharmaceuticals.

[40] Boukamp P., Petrussevska R.T., Breitkreutz D., Hornung J., Markham A., Fusenig N.E. (1988). Normal Keratinization in a Spontaneously Immortalized Aneuploid Human Keratinocyte Cell Line. J. Cell Biol..

[41] Goff P.S., Castle J.T., Kohli J.S., Sviderskaya E.V., Bennett D.C. (2023). Isolation, Culture, and Transfection of Melanocytes. Curr. Protoc..

[42] Nair G., Hema Sree G.N.S., Saraswathy G.R., Marise V.L.P., Krishna Murthy T.P. (2021). Application of comprehensive bioinformatics approaches to reconnoiter crucial genes and pathways underpinning hepatocellular carcinoma: A drug repurposing endeavor. Med. Oncol..

[43] Kerr J.F.R., Wyllie A.H., Currie A.R. (1972). Apoptosis: A basic biological phenomenon with wide-ranging implications in tissue kinetics. Br. J. Cancer.

[44] Wrobel L.J., Le Gal F.A. (2015). Inhibition of human melanoma growth by a non-cardioselective β-blocker. J. Investig. Dermatol..

[45] Suzuki-Karasaki Y., Fujiwara K., Saito K., Suzuki-Karasaki M., Ochiai T., Soma M. (2015). Distinct effects of TRAIL on the mitochondrial network in human cancer cells and normal cells: Role of plasma membrane depolarization. Oncotarget.

[46] Borenfreund E., Babich H., Martin-Alguacil N. (1988). Comparisons of two in vitro cytotoxicity assays—The neutral red (NR) and tetrazolium MTT tests. Toxicol. In Vitr..

[47] Fotakis G., Timbrell J.A. (2006). In vitro cytotoxicity assays: Comparison of LDH, neutral red, MTT and protein assay in hepatoma cell lines following exposure to cadmium chloride. Toxicol. Lett..

[48] Saha J., Kim J.H., Amaya C.N., Witcher C., Khammanivong A., Korpela D.M., Brown D.R., Taylor J., Bryan B.A., Dickerson E.B. (2021). Propranolol Sensitizes Vascular Sarcoma Cells to Doxorubicin by Altering Lysosomal Drug Sequestration and Drug Efflux. Front. Oncol..

[49] Smiley S.T., Reers M., Mottola-Hartshorn C., Lin M., Chen A., Smith T.W., Steele G.D., Chen L.B. (1991). Intracellular heterogeneity in mitochondrial membrane potentials revealed by a J-aggregate-forming lipophilic cation JC-1. Proc. Natl. Acad. Sci. USA.

[50] Perelman A., Wachtel C., Cohen M., Haupt S., Shapiro H., Tzur A. (2012). JC-1: Alternative excitation wavelengths facilitate mitochondrial membrane potential cytometry. Cell Death Dis..

[51] Sim G., Ok S.H., Lee S.H., Park K.E., Park S., Sohn J.T. (2025). Lipid Emulsion Mitigates the Cardiotoxic Effects of Labetalol in Rat Cardiomyoblasts. Cells.

[52] Kozanoglu I., Yandim M.K., Cincin Z.B., Ozdogu H., Cakmakoglu B., Baran Y. (2013). New indication for therapeutic potential of an old well-known drug (propranolol) for multiple myeloma. J. Cancer Res. Clin. Oncol..

[53] Pendergrass W., Wolf N., Poot M. (2004). Efficacy of MitoTracker Green and CMXRosamine to measure changes in mitochondrial membrane potentials in living cells and tissues. Cytom. Part A.

[54] Desai S., Grefte S., van de Westerlo E., Lauwen S., Paters A., Prehn J.H.M., Gan Z., Keijer J., Adjobo-Hermans M.J.W., Koopman W.J.H. (2024). Performance of TMRM and MitoTrackers in mitochondrial morphofunctional analysis of primary human skin fibroblasts. Biochim. Biophys. Acta–Bioenerg..

[55] Serasinghe M.N., Wieder S.Y., Renault T.T., Elkholi R., Asciolla J.J., Yao J.L., Jabado O., Hoehn K., Kageyama Y., Sesaki H. (2015). Mitochondrial division is requisite to RAS-induced transformation and targeted by oncogenic MAPK pathway inhibitors. Mol. Cell.

[56] Brohée L., Peulen O., Nusgens B., Castronovo V., Thiry M., Colige A.C., Deroanne C.F. (2018). Propranolol sensitizes prostate cancer cells to glucose metabolism inhibition and prevents cancer progression. Sci. Rep..

[57] Valente A.J.F., Maddalena L.A., Robb E.L., Moradi F., Stuart J.A. (2017). A simple ImageJ macro tool for analyzing mitochondrial network morphology in mammalian cell culture. Acta Histochem..

[58] Okazawa M., Shiraki T., Ninomiya H., Kobayashi S., Masaki T. (1998). Endothelin-induced apoptosis of A375 human melanoma cells. J. Biol. Chem..

[59] Moss D.K., Betin V.M., Malesinski S.D., Lane J.D. (2006). A novel role for microtubules in apoptotic chromatin dynamics and cellular fragmentation. J. Cell Sci..

[60] Niero E.L.O., Machado-Santelli G.M. (2013). Cinnamic acid induces apoptotic cell death and cytoskeleton disruption in human melanoma cells. J. Exp. Clin. Cancer Res..

[61] Solernó L.M., Sobol N.T., Gottardo M.F., Capobianco C.S., Ferrero M.R., Vásquez L., Alonso D.F., Garona J. (2022). Propranolol blocks osteosarcoma cell cycle progression, inhibits angiogenesis and slows xenograft growth in combination with cisplatin-based chemotherapy. Sci. Rep..

[62] Li P., Nijhawan D., Budihardjo I., Srinivasula S.M., Ahmad M., Alnemri E.S., Wang X. (1997). Cytochrome c and dATP-dependent formation of Apaf-1/caspase-9 complex initiates an apoptotic protease cascade. Cell.

[63] Brentnall M., Rodriguez-Menocal L., Ladron De Guevara R., Cepero E., Boise L.H. (2013). Caspase-9, caspase-3 and caspase-7 have distinct roles during intrinsic apoptosis. BMC Cell Biol..

[64] Farhoumand L.S., Liu H., Tsimpaki T., Hendgen-Cotta U.B., Berchner-Pfannschmidt U. (2023). Blockade of β-adrenergic receptors by nebivolol enables tumor control potential for uveal melanoma in 3D tumor spheroids and 2D cultures. Int. J. Mol. Sci..

[65] Ribble D., Goldstein N.B., Norris D.A., Shellman Y.G. (2005). A simple technique for quantifying apoptosis in 96-well plates. BMC Biotechnol..

[66] Kasibhatla S., Amarante-Mendes G.P., Finucane D., Brunner T., Bossy-Wetzel E., Green D.R. (2006). Propidium Iodide (PI) Uptake Assay to Detect Apoptosis. Cold Spring Harb. Protoc..

[67] Chen J., Huang C., Liu F., Xu Z., Li L., Huang Z., Zhang H. (2019). Methylwogonin exerts anticancer effects in A375 human malignant melanoma cells through apoptosis induction, DNA damage, cell invasion inhibition and downregulation of the mTOR/PI3K/Akt signalling pathway. Arch. Med. Sci..

[68] Bustamante P., Miyamoto D., Goyeneche A., de Alba Graue P.G., Jin E., Tsering T., Dias A.B., Burnier M.N., Burnier J.V. (2019). Beta-blockers exert potent anti-tumor effects in cutaneous and uveal melanoma. Cancer Med..

[69] Viera L.M.A.L., Silva R.S., da Silva C.C., Presgrave O.A.F., Boas M.H.S.V. (2022). Comparison of the different protocols of the Hen’s Egg Test-Chorioallantoic Membrane (HET-CAM) by evaluating the eye irritation potential of surfactants. Toxicol. In Vitr..

[70] Puenea G., Almăjan-Guță B., Chioibaș R., Macașoi I., Geamantan A., Dinu S., Mergheș P., Boia E.R., Tischer A.A., Iftode A. (2023). Evaluation of oleanolic acid, doxorubicin and their association in the treatment of melanoma: Enhanced efficacy and antiangiogenic potential. Farmacia.

[71] Faur A., Watz C., Moacă E.-A., Avram Ş., Borcan F., Pinzaru I., Iftode A., Nicolov M., Popovici R.A., Raica M. (2020). Correlations on Phenolic Screening Related to In Vitro and In Ovo Assessment of Ocimum basilicum L. Hydro-Alcoholic Extracts Used as Skin Active Ingredient. Molecules.

[72] Rivero M.N., Lenze M., Izaguirre M., Pérez Damonte S.H., Aguilar A., Wikinski S., Gutiérrez M.L. (2021). Comparison between HET-CAM protocols and a product use clinical study for eye irritation evaluation of personal care products including cosmetics according to their surfactant composition. Food Chem. Toxicol..

[73] Breban-Schwarzkopf D., Danila A.I., Tanase A.D., Munteanu K., Haj Ali D., Popovici R., Szuhanek C., Rominu M. (2024). Eugenol—A natural alternative in dentistry: An in vitro and in ovo biosafety assessment. Farmacia.

[74] Balestrin L.A., Kreutz T., Fachel F.N.S., Bidone J., Gelsleichter N.E., Koester L.S., Bassani V.L., Braganhol E., Dora C.L., Teixeira H.F. (2021). *Achyrocline satureioides* (Lam.) DC (*Asteraceae*) Extract-Loaded Nanoemulsions as a Promising Topical Wound Healing Delivery System: In Vitro Assessments in Human Keratinocytes (HaCaT) and HET-CAM Irritant Potential. Pharmaceutics.

[75] Romashin D., Rusanov A., Arzumanian V., Varshaver A., Poverennaya E., Vakhrushev I., Netrusov A., Luzgina N. (2024). Exploring the Functions of Mutant p53 through *TP53* Knockout in HaCaT Keratinocytes. Curr. Issues Mol. Biol..

